# The NLRP3 Inflammasome and IL-1β Accelerate Immunologically Mediated Pathology in Experimental Viral Fulminant Hepatitis

**DOI:** 10.1371/journal.ppat.1005155

**Published:** 2015-09-14

**Authors:** Sheng Guo, Chengying Yang, Bo Diao, Xiaoyong Huang, Meihua Jin, Lili Chen, Weiming Yan, Qin Ning, Lixin Zheng, Yuzhang Wu, Yongwen Chen

**Affiliations:** 1 Institute of Immunology, PLA, Third Military Medical University, Chongqing, China; 2 Department of Pharmacology, Yanbian University, Yanji, Jilin province, China; 3 Department of Basic Medicine, Yanbian University, Yanji, Jilin province, China; 4 Department and Institute of Infectious Disease, Tongji Hospital, Tongji Medical College, Huazhong University of Science and Technology, Wuhan, China; 5 Laboratory of Immunology, National Institute of Allergy and Infectious Diseases, National Institutes of Health, Bethesda, Maryland, United States of America; Nationwide Children's Hospital, UNITED STATES

## Abstract

Viral fulminant hepatitis (FH) is a severe disease with high mortality resulting from excessive inflammation in the infected liver. Clinical interventions have been inefficient due to the lack of knowledge for inflammatory pathogenesis in the virus-infected liver. We show that wild-type mice infected with murine hepatitis virus strain-3 (MHV-3), a model for viral FH, manifest with severe disease and high mortality in association with a significant elevation in IL-1β expression in the serum and liver. Whereas, the viral infection in IL-1β receptor-I deficient (*IL-1R1* ^*-/-*^) or IL-1R antagonist (IL-1Ra) treated mice, show reductions in virus replication, disease progress and mortality. *IL-1R1* deficiency appears to debilitate the virus-induced fibrinogen-like protein-2 (FGL2) production in macrophages and CD45^+^Gr-1^high^ neutrophil infiltration in the liver. The quick release of reactive oxygen species (ROS) by the infected macrophages suggests a plausible viral initiation of NLRP3 inflammasome activation. Further experiments show that mice deficient of *p47* ^*phox*^, a nicotinamide adenine dinucleotide phosphate (NADPH) oxidase subunit that controls acute ROS production, present with reductions in NLRP3 inflammasome activation and subsequent IL-1β secretion during viral infection, which appears to be responsible for acquiring resilience to viral FH. Moreover, viral infected animals in deficiencies of NLRP3 and Caspase-1, two essential components of the inflammasome complex, also have reduced IL-1β induction along with ameliorated hepatitis. Our results demonstrate that the ROS/NLRP3/IL-1β axis institutes an essential signaling pathway, which is over activated and directly causes the severe liver disease during viral infection, which sheds light on development of efficient treatments for human viral FH and other severe inflammatory diseases.

## Author Summary

The NLRP3 inflammasome and IL-1β play essential roles in mediating the primary inflammatory responses against pathogen invasions in the host. Hyperactivation of this signaling pathway can lead to life-threatening diseases under certain circumstances. However, it is not clear if NLRP3 inflammasome activation participates in the pathogenesis of viral fulminant hepatitis (FH), a clinical severe syndrome characterized by acute inflammation in the liver along with massive necrosis of hepatocytes and hepatic encephalopathy during viral infection. Using a mouse viral FH model by infection with murine hepatitis virus strain-3 (MHV-3), we observed a significant macrophage induction and the serum and liver massive accumulation of IL-1β. Conversely, interruption of IL-1β signals results in attenuation of the MHV-3-induced hepatitis and mortality. Blocking IL-1β activity reduces the virus-induced expression of fibrinogen-like protein-2 (FGL2) in macrophages, and limits the liver recruitment of CD45^+^Gr-1^high^ neutrophils upon the virus infection. We further show that proIL-1β is bioprocessed by NLRP3 inflammasome. Deletion of the components in the inflammasome complex, including *NLRP3* and *Caspase-1*, leads to reduction in the virus-induced IL-1β production and lessening of disease progression. Further studies show that macrophages in deficiency of nicotinamide adenine dinucleotide phosphate (NADPH) oxidase subunit *p47* ^*phox*^, a protein that controls acute ROS production, prevents NLRP3 inflammasome activation and IL-1β secretion, suggesting that the virus-induced ROS production can directly initiate NLRP3 inflammasome activation. Therefore, *p47* ^*phox-/-*^ mice exhibited certain degrees of MHV-3 resistance. Taken together, these results demonstrate that ROS/NLRP3/IL-1β is the key pathway signaling exacerbated inflammatory responses that cause viral FH in mice, suggesting that mediation of this signal cascade may benefit on the disease treatment.

## Introduction

Viral fulminant hepatitis (FH) is a clinical syndrome characterized by massive necrosis of hepatocytes along with hepatic encephalopathy during the infections [1]. Despite advances in the development of antiviral drugs, a poor understanding of the immune mechanisms underlying viral FH has largely stalled the identification of effective clinical interventions. Fortunately, the recent development of an animal model of FH using murine hepatitis virus strain-3 (MHV-3) infection has provided insights in understanding the pathogenesis and developing novel therapeutics for the disease [2].

MHV-3 is a single-stranded, positive-sense RNA virus belonging to the coronavirus family [3]. The hallmarks of MHV-3-induced FH in susceptible BALB/cJ and C57BL/6 mice include the appearance of liver sinusoidal thrombosis and hepatocellular necrosis, resulting from over expression of a virus-induced, monocyte/macrophage-specific procoagulant, fibrinogen-like protein-2 (FGL2). Liver accumulation of FGL2 directly activates the coagulation cascades, a phenomenon known as virus induced procoagulant activity [3]. MHV-3-induced FH exhibits a syndrome that is very similar to the clinical manifestations of patients with viral FH, making it a good animal model for exploring mechanisms underlying the pathogenesis of human viral FH.

In addition to FGL2, pro-inflammatory mediators such as TNF-α, IFN-γ and complement C5a have been proposed to accelerate viral FH pathogenesis [4, 5]. Nevertheless, the mechanisms on how the inflammatory signaling events that regulate the disease progression are not well understood. Recently, it has been shown that dysregulated NLRP3 (also known as NALP3 and cryopyrin) inflammasome in macrophages causes the pathogenesis of inflammatory diseases, which highlights the importance of inflammasome in regulating immune-mediated tissue damages [6]. The generation of biologically active IL-1β requires cleavage of the inactive precursor proIL-1β by the NLRP3 inflammasome, a protein-scaffolding complex consisting of NLRP3, Caspase-1, and the adaptor molecule ASC (apoptosis-associated peck-like protein with CARD domain, Pycard) [6, 7]. NLRP3 inflammasome and IL-1β mediate the host protection against pathogen invasions, whereas, the hyperactivation of NLRP3 inflammasome contributes to the pathogenesis of certain inflammatory syndromes, including liver injuries such as nonalcoholic/alcoholic steatohepatitis [8, 9], liver fibrosis [10], and immune mediated liver injuries [11]. However, the role of NLRP3 inflammasome signaling pathway participates in the pathogenesis of viral FH is still unclear.

A variety of danger-associated molecular patterns (DAMPs) and pathogen-associated molecular patterns (PAMPs), including virus RNA, nigericin, ATP, silica crystals, mitochondrial DNA, and aluminum hydroxide, appear to be capable of activating the NLRP3 inflammasome [12]. Nevertheless, the reactive oxygen species (ROS) generated by nicotinamide adenine dinucleotide phosphate (NADPH) oxidase are considered to be one of the major factors that activate NLRP3 inflammasome [13]. It has been shown that pharmacological inhibition of the NADPH oxidase complex (NOX) or the down regulation of the NOX subunit *p22* ^*phox*^ eliminates NLRP3 inflammasome activation by preventing ROS secretion [13, 14]. However, recent studies have also illustrated that mitochondria-originated ROS (MitoSOX) rather than NOX-derived ROS drive NLRP3 inflammasome activation [15, 16]. Various stress condition, including increased metabolic rates, hypoxia, or membrane damage, all significantly induce MitoSOX secretion [17]. Conversely, it remains uncertain for which of the NOX-derived ROS or MitoSOX is responsible for causing NLRP3 inflammasome- dependent pathology in viral FH development.

Here, we showed that C57BL/6 wild type (WT) mice infected with MHV-3 manifest with high levels of IL-1β in the serum and liver. Conversely, the virus infected *IL-1R1* ^*-/-*^ mice present with much attenuated pathologies, showing with a significant reduction in macrophage-derived FGL2 expression and less liver infiltration of CD45^+^Gr-1^high^ neutrophils. Furthermore, we showed that the *in vivo* bioactivation of proIL-1β during MHV-3 infection is mediated by NLRP3 inflammasome activation, thereafter, both the *NLRP3* ^*-/-*^ mice and the *Caspase-1* ^*-/-*^ mice display substantial resistance to MHV-3-induced IL-1β production. Mechanistically, MHV-3 infection triggers an acute release of NOX-derived ROS. Blocking ROS with Diphenyleneiodonium chloride (DPI) inhibits Caspase-1 activation and IL-1β maturation *in vitro*. Furthermore, NOX subunit *p47* ^*phox*^- deficient mice also exhibited a delayed and moderate viral pathogenesis due to reduction in NLRP3 inflammasome activation *in vivo*. These results reveal that the ROS/NLRP3/IL-1β axis is a critical signaling pathway leading to the pathogenesis of viral FH.

## Results

### Excessive IL-1β production in viral fulminant hepatitis

**Fig 1:**
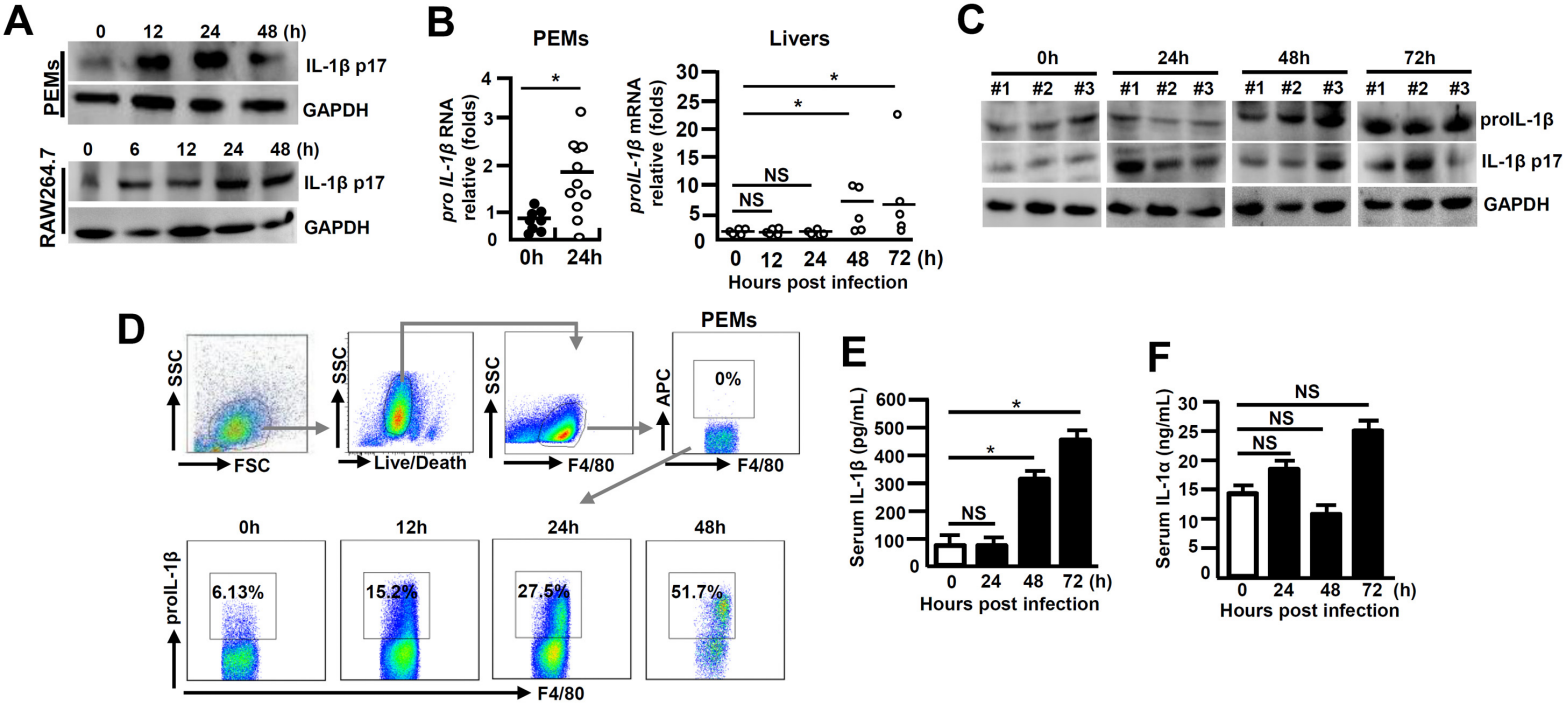
Augmented IL-1β expression during viral FH. **(A)** Cultured peritoneal exudative macrophages (PEMs) and RAW264.7 cells were infected with MHV-3 (MOI = 1) *in vitro*, and IL-1β p17 protein levels were detected in the indicated time points by western-blotting. C57BL/6 WT mice were infected with MHV-3 (100PFU), **(B)** PEMs and livers were isolated and the transcription of *proIL-1β* mRNA was measured by qPCR. *p<0.05, NS: no significant difference. **(C)** The expression of proIL-1β and IL-1β p17 in virus-infected liver tissues was detected by western-blotting. Three representative samples *per* group are shown. **(D)** The proIL-1β protein in PEMs isolated from MHV-3 infected mice at the indicated time points was detected by flow cytometry. The up panels are gate strategies and number indicates the percentage of positive cells in the gate. One representative sample from five mice *per* group is showed. **(E)** Serum IL-1β and **(F)** IL-1α levels in virus-infected mice were detected by ELISA. N = 5~10 *per* group,*p<0.05, NS: no significant difference.

To examine the status of IL-1β activation in macrophages in response to MHV-3 infection, primary peritoneal exudative macrophages (PEMs) and the macrophage line-RAW264.7 cells were infected with the virus *in vitro*. A time course data showed a significant induction of the activated form of IL-1β (IL-1β p17) within 12 hours, sustaining to 48h (Fig 1A). Assessment of the PEMs isolated from the 24h of virus infected C57BL/6 WT mice also revealed a significant increase in *proIL-1β* mRNA expression (Fig 1B). Moreover, *proIL-1β* mRNA expression in the infected livers appeared to be markedly augmented at 48h (*p* = 0.0231), sustaining to 72h (*p* = 0.0356, Fig 1B). In accordance, western-blotting showed with increases in proIL-1β and IL-1β p17 protein expression at corresponding time points in the infected livers (Fig 1C). Flow cytometry further validated the patterns of proIL-1β protein induction in the PEMs isolated from the virus-infected mice (Fig 1D). In agreement, the infected mice also showed significant accumulation of serum IL-1β during the infection (Fig 1E). In contrast, serum IL-1α concentration exhibited little change in MHV-3 infected mice (Fig 1F). These results suggest that IL-1β significantly elevate in the liver and periphery during viral FH.

### Intervention of IL-1β signaling reduces MHV-3-mediated hepatitis

**Fig 2:**
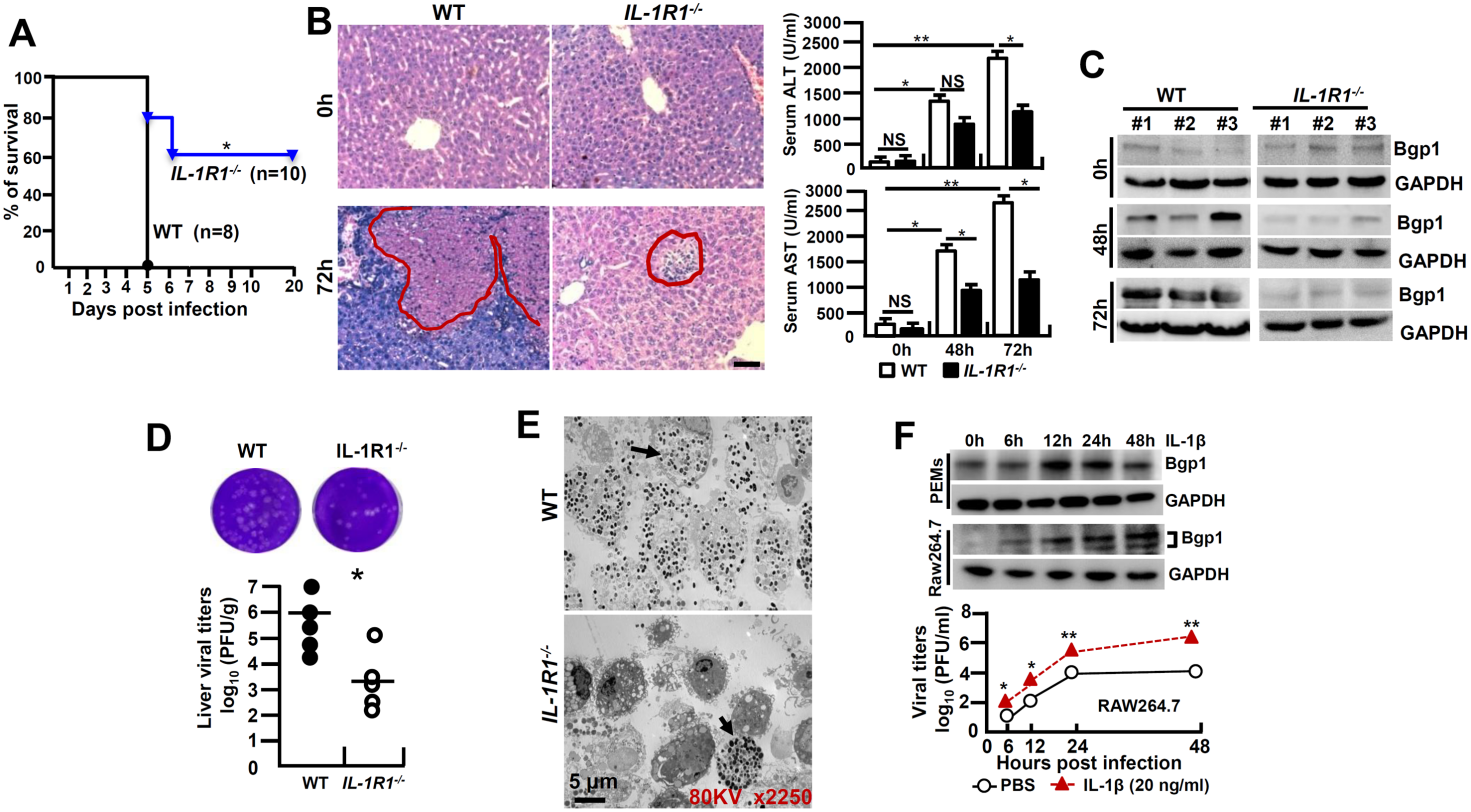
*IL-1R1* deficiency attenuates MHV-3-induced hepatitis. *IL-1R1* ^*-/-*^ mice and their C57BL/6 wild-type (WT) littermates were infected with MHV-3 (100 PFU), **(A)** The survival rate was monitored for a total of 20 days. One representative of three experiments with similar results is shown. *p<0.05. **(B)** The liver architecture was analyzed by H&E staining (left), and serum ALT/AST levels were determined with an AU5400 automatic biochemistry analyzer (right). Scale bar 20 μm, n = 5 *per* group, *p<0.05 and **p<0.001, NS: no significant difference. **(C)** The expression of Bgp1 in MHV-3-infected livers was compared by western-blotting. Three representative samples *per* group are shown. **(D)** The virus titers in livers at 72h post-infection were analyzed by plaque assay (up), and results were compared by statistical analysis (down). *p<0.05. (**E**) Peritoneal exudative macrophages (PEMs) were isolated from virus infected mice at 24h and the virions were detected by electron microscopy. Arrows indicate spherical virions. **(F)** The expression of Bgp1 in PEMs and RAW264.7 cells that treated with IL-1β (20 ng/ml) at the indicated time points was detected by western-blotting (up panel). RAW264.7 cells were treated with IL-1β (20 ng/ml) and PBS for a total 48h firstly, cells were further infected with MHV-3 and virus titers were detected by plaque assay at the indicated time points (down panel). *p<0.05 compared to PBS-treated counterparts.

IL-1β amplifies the pro-inflammatory response *via* the type-I of IL-1 receptor (*IL-1R1*) [18]. To further investigate whether IL-1β signaling affects the pathogenesis of viral FH, we infected *IL-1R1* ^*-/-*^ mice with MHV-3 (100 PFU) *via* intraperitoneal (i.p.) injection. Interestingly, *IL-1R1* ^*-/-*^ mice displayed with a significant increase in survival rate with 60% staying alive for 20 days, as compared to a 100% death of the WT littermates within 5 days of the viral infection (Fig 2A). *IL-1R1* ^*-/-*^ mice manifested a significant reduction in hepatocellular damage and a decrease in serum ALT/AST levels during the infection (Fig 2B). The expression of biliary glycoprotein-1 (Bgp1), the receptor for MHV-3 [19], appeared to be significantly lower in the virus infected *IL-1R1* ^*-/-*^ livers comparing to that in the WT controls (Fig 2C), concurring with the plaque assay data showing with limited virus entrance and amplification in the livers 72h post-infection (Fig 2D). In support, the MHV-3 infection efficiency in *IL-1R1* ^*-/-*^ PEMs dropped more significantly than in the WT counterparts *in vivo* (Fig 2E). Obviously, recombinant mouse IL-1β protein (20 ng/ml) is able to significantly induce Bgp1 expression in PEMs and RAW264.7 cells *in vitro* (Fig 2F), and in concurrence, IL-1β treated RAW264.7 cells appear to produce more virus than the PBS treated controls post-infection (Fig 2F). In validation, we injected the virus-infected WT mice with IL-1R antagonist (IL-1Ra, 10 mg/kg/day), a naturally occurring cytokine that blocks IL-1β biologic response [18], and observed a significant limitation of IL-1β secretion (*p* = 0.0007, S1A Fig), inhibition of Bgp1 expression (S1B Fig) and reduction of virus titers (S1C Fig), suggesting the existence of an IL-1R-dependent positive regulation on the virus receptor that directly associate with virus propagation in the host. These combined data clearly demonstrate that IL-1β promotes viral amplification and exacerbates the progression of hepatitis.

### MHV-3 fails to induce FGL2 production and liver neutrophil infiltration in *IL-1R1* ^*-/-*^ mice

**Fig 3:**
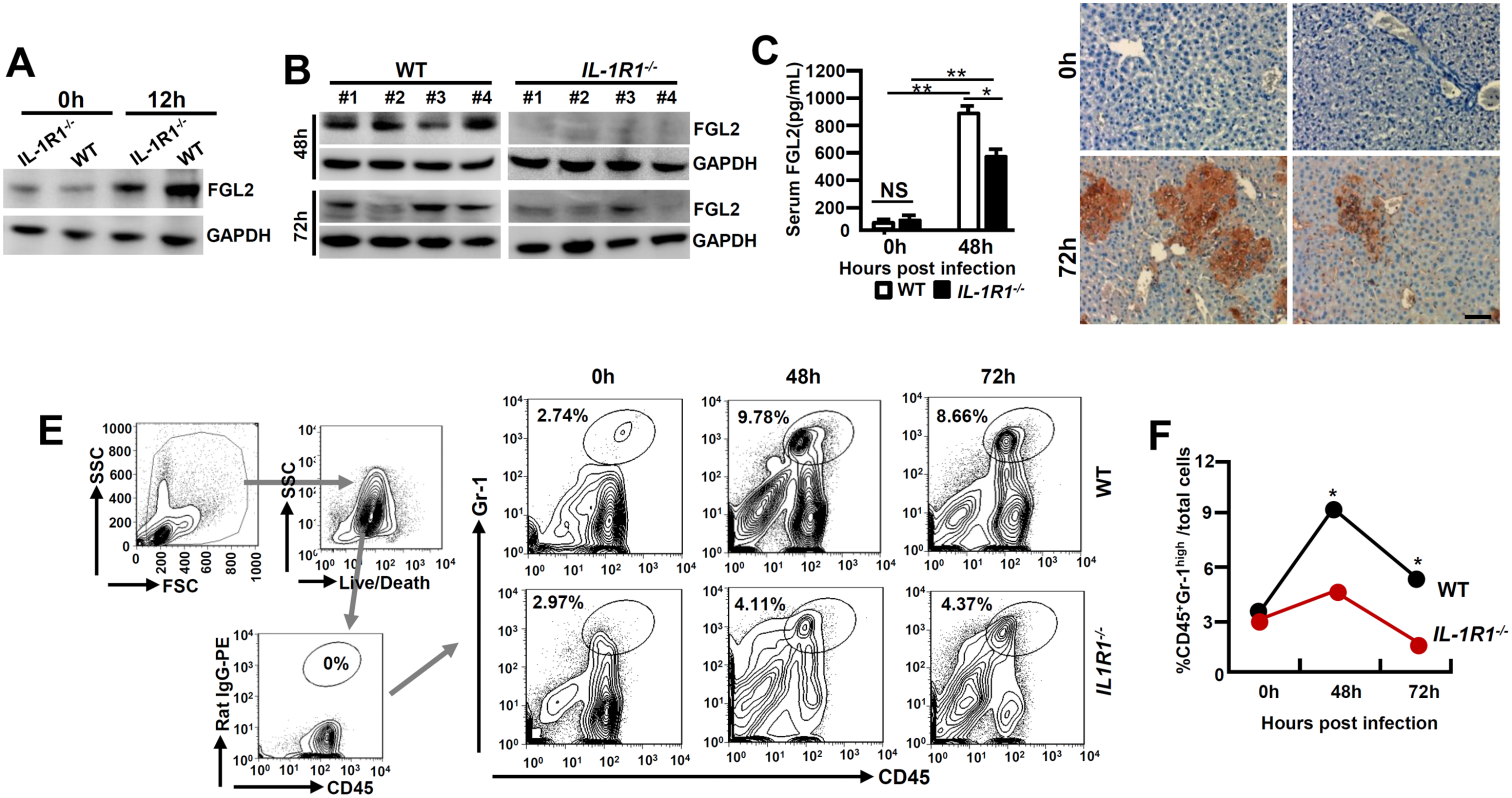
MHV-3 fails to induce FGL2 production and neutrophil infiltration in the livers of *IL-1R1* ^*-/-*^ mice. *IL-1R1* ^*-/-*^ mice and their C57BL/6 WT littermates were infected with MHV-3 (100 PFU). **(A)** Peritoneal exudative macrophages (PEMs) were isolated and the expression of FGL2 was detected by western-blotting. **(B)** The expression of FGL2 in liver at 48h and 72h post-infection was analyzed by western-blotting. Four representative samples *per* group are shown. **(C)** Serum FGL2 levels in virus infected mice were measured by ELISA.*p<0.05 and **p<0.0001, NS: no significant difference, n = 5 *per* group. **(D)** The liver fibrinogen deposition post-infection was analyzed by immunohistochemistry. Scale bar 20 μm, n = 6~8 *per* group. **(E)** Liver recruitment of CD45^+^Gr-1^high^ neutrophils after MHV-3 infection was measured by flow cytometry. The left panels are gate strategies, and number indicates the percentage of positive cells in the gate. One representative sample from five mice *per* group is showed. **(F)** Statistical analysis of liver CD45^+^Gr-1^high^ neutrophil infiltration. *p<0.05 compared to WT littermates in each group, n = 5 *per* group.

FGL2 plays an essential role in inducing hepatocellular necrosis following MHV-3 infection [3]. We firstly examined FGL2 expression in PEMs isolated from MHV-3 infected *IL-1R1* ^*-/-*^ mice and observed substantial lower levels of FGL2 as compared to the WT controls (Fig 3A). The limited FGL2 expression in macrophages correlates with the low concentrations of FGL2 observed in the virus infected *IL-1R1* ^*-/-*^ liver and serum (Fig 3B and 3C). Therefore, in response to MHV-3 viral infection, *IL-1R1* ^*-/-*^ mice responded with limited fibrinogen formation, leading to a down modulation of liver coagulation and necrosis (Fig 3D). Similarly, IL-1Ra-treated WT mice displayed with reduction of FGL2 and fibrinogen deposition in liver tissues, which was followed with decrease in liver damages and enhance the survival time (S1B, S1D and S1E Fig).

Neutrophils and CD4^+^Foxp3^+^ regulatory T cells (Tregs) have been well recognized as important players in viral FH [20, 21]. To determine the role of IL-1β in regulating these cells during viral FH, we firstly examined liver neutrophil infiltration status. Flow cytometry showed that in the liver-tissue samples from 48 and 72h post MHV-3 infection, the infiltration of CD45^+^Gr-1^high^ neutrophils was substantially higher in the WT livers than that in the *IL-1R1* ^*-/-*^ littermates (Fig 3E and 3F). The number of CD4^+^Foxp3^+^Treg in the virus-infected livers appeared to increase significantly after MHV- 3 infection, nevertheless, little difference was observed between *IL-1R1* ^-/-^ mice and their WT controls (S2 Fig). Similarly, serum concentration of C5a, a cytokine that deteriorates the pathogenesis of MHV-3-mediated FH [5], was not changed dramatically between virus infected *IL-1R1* ^*-/-*^ mice and their WT controls (S3A Fig). These results suggest that attenuation of viral FH by *IL-1R1* deficiency could be the consequence of both ineffective FGL2 production by macrophages and limited CD45^+^Gr-1^high^ neutrophil infiltration in the affected liver.

### IL-1β and TNF-α synergistically activate NF-κB for FGL2 induction in macrophages

**Fig 4:**
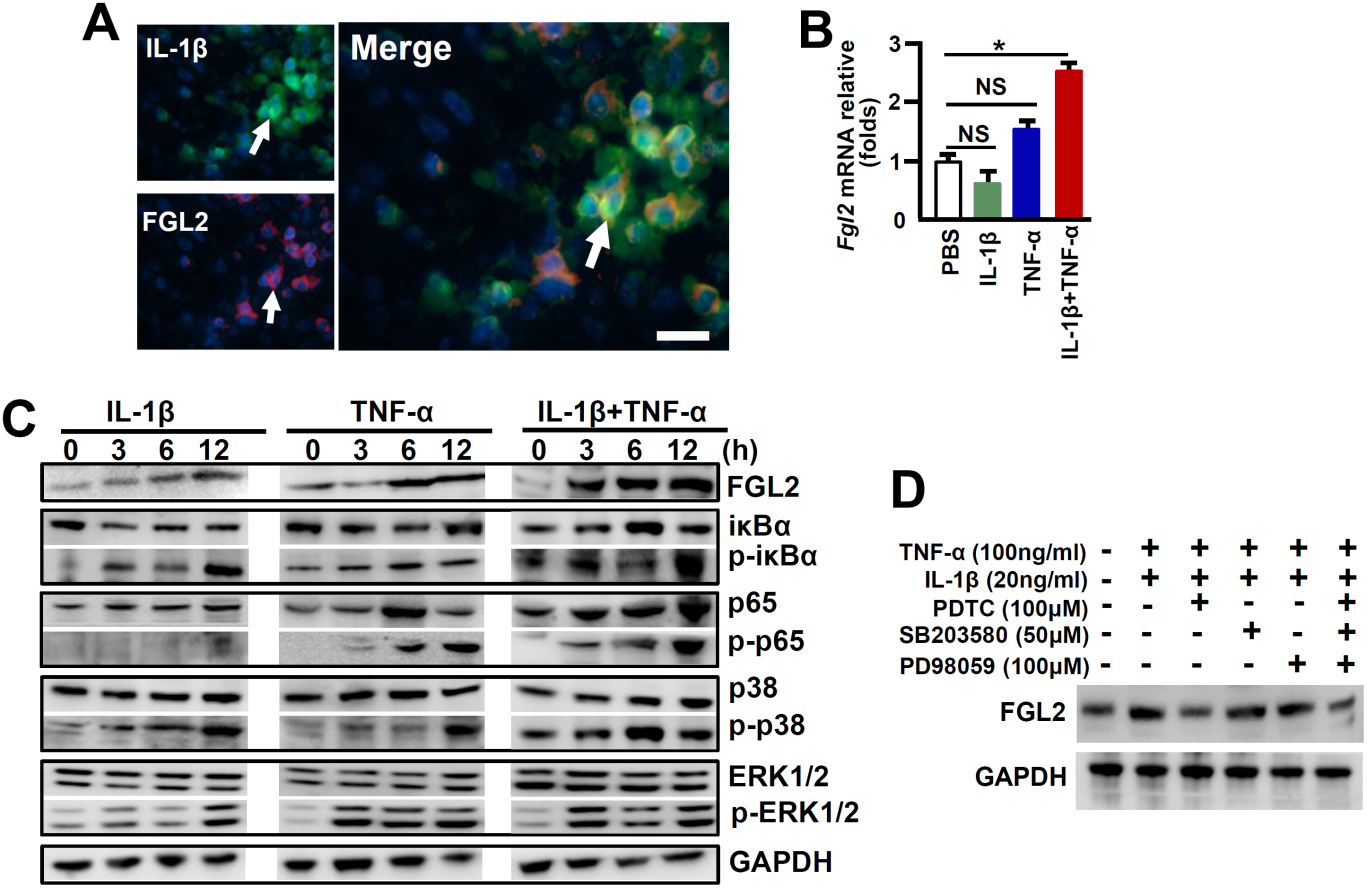
IL-1β synergistically acts with TNF-α to promote FGL2 overexpression through activation of NF-κB signaling. **(A)** The expression of IL-1β-p17 and FGL2 was detected by immunofluorensent double staining. Arrows indicate positive cells, blue color indicates nuclear staining with 4',6-diamidino-2-phenylindole (DAPI), scale bar 20 μm. RAW264.7 cells were treated with IL-1β (20 ng/ml), TNF-α (100 ng/ml) alone or in combination, **(B)** The transcription of *fgl2* at 24h post-cytokine treatment was detected by qPCR. *p<0.05, NS: no significant difference, one of three experiments with similar results is shown. **(C)** Immunoblot analysis of phosphorylated (p-) and total signaling proteins in whole-cell lysates of RAW264.7 cells stimulated for various times with IL-1β, TNF-α alone or in the combination. **(D)** RAW264.7 cells were treated with IL-1β/TNF-α for a total of 24h, and cells were further incubated with inhibitors against NF-κB (PDTC), ERK (PD98059) and p38-MAPK (SB203580) in the last 6h, the expression of FGL2 was detected by western-blotting. One of three experiments with similar results is shown.

A reduction of FGL2 expression was observed in *IL-1R1* ^*-/-*^ mice in response to MHV-3 infection, together with IL-1β and FGL2 were co-expression in PEMs (Fig 4A), implying that IL-1β/IL-1R1 interactions may directly regulate FGL2 expression in macrophages. To address the issue, we treated RAW264.7 cells, a macrophage line capable of expressing FGL2, with the recombinant mouse IL-1β protein (20 ng/ml) *in vitro*. qPCR and western-blotting data showed that IL-1β alone is incapable of stimulating FLG2 expression, nevertheless, it synergistically enhances TNF-α-induced FGL2 levels (Fig 4B and 4C).

The expression of FGL2 has been proposed to be mediated through the activation of NF-κB and mitogen-activated protein kinase (MAPK) signaling pathways under inflammatory conditions [5, 22]. To further investigate the molecular mechanisms through which IL-1β promotes FGL2 production, we examined these signaling pathways in IL-1β-treated RAW264.7 cells. Results showed that either IL-1β or TNF-α treatment alone, had a minimum stimulation on phosphorylation of the NF-κB chaperone IκBα (p-IκBα) and the NF-κB subunit p65 (p-p65), appearing only at extended incubation time point (12h). However, synergistic effects of IL-1β and TNF-α (IL-1β+TNF-α) seemed to be significant for which substantial increases in phosphorylation of IκBα and p65 can be detected as early as 3h post infection (Fig 4C). Furthermore, the inhibition of NF-κB activation by Pyrrolidinedithiocarbamic Acid (PDTC) successfully prevented FGL2 upregulation after IL-1β+TNF-α treatment (Fig 4D). The combination of IL-1β and TNF-α is capable of potently stimulation the phosphorylation of MAPKs, including extracellular signal-related kinase (p-ERK1/2) and p38 (pp38) (Fig 4C). Nevertheless, the ERK inhibitor-PD98059 and the p38-MAPK inhibitor-SB203580 seemed to be incapable of blocking FGL2 upregulation. Moreover, blocking all of these three pathways did not show additive effect on inhibition of FGL2 expression (Fig 4D). These results suggest that NF-κB rather than the MAPK pathways is responsible for IL-1β+TNF-α-mediated FGL2 upregulation in viral infected macrophages.

### MHV-3 stimulates NLRP3 inflammasome-dependent IL-1β activation

**Fig 5:**
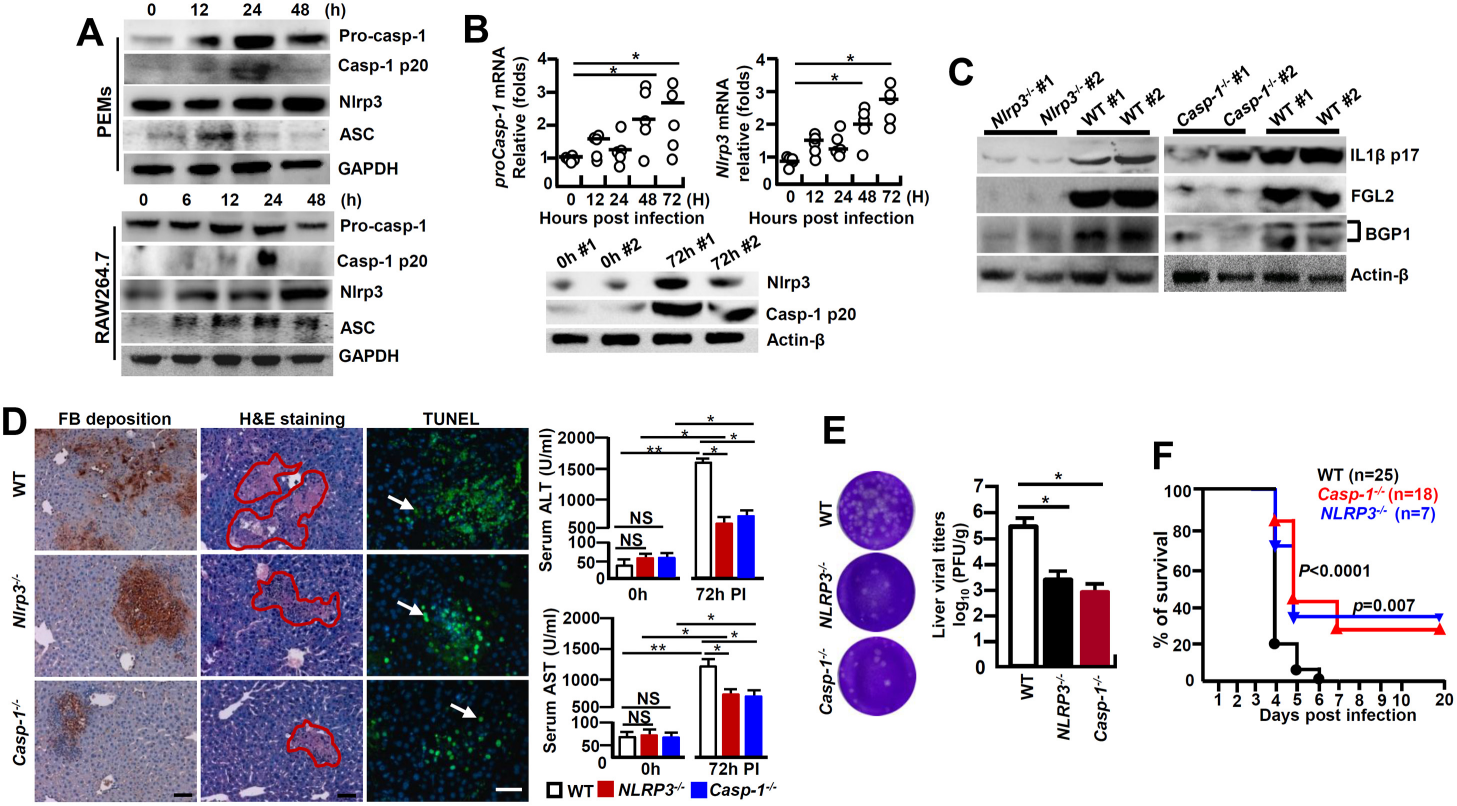
NLRP3 inflammasome involves in regulating the pathogenesis of MHV-3-mediated hepatitis. **(A)** Peritoneal exudative macrophages (PEMs) and RAW264.7 cells were infected with MHV-3 (MOI = 1) *in vitro*, and the expression of NLRP3 inflammasome complex components in the indicated time points was detected by western-blotting. **(B)** The transcription of *NLRP3* and *proCaspase-1* mRNA in liver tissues isolated from MHV-3 infected C57BL/6 WT mice was detected by qPCR (up penal), and the protein levels were measured by western-blotting (down penal). *p<0.05, NS: no significant difference. **(C)** The expression of IL-1β-p17, FGL2 and Bgp1 in *NLRP3* ^*-/-*^, *Caspase-1* ^*-/-*^ and C57BL/6 WT livers at 0h and 72h post-infection was measured by western-blotting. **(D)** Liver fibrinogen deposition was analyzed by immunohistochemistry, the architecture was analyzed by H&E staining and cellular apoptosis was analyzed using TUNEL staining (left). Arrow indicates positive cells, blue color indicates nuclear staining with DAPI, scale bar 20 μm, n = 5 *per* group. Serum ALT and AST activities were determined with an AU5400 automatic biochemistry analyzer (right).*p<0.05, ** *p*<0.001, n = 5 *per* group, NS: no significant difference. **(E)** The virus titers in livers at 72h post-infection were analyzed by plaque assay (left), and their levels were compared by statistical analysis (right). *p<0.05, n = 5 *per* group. **(F)** The survival rate of virus infected mice was monitored for a total of 20 days. One representative of four experiments with similar results is shown. *p*<0.05 was considered significant different.

It has been established that the Caspase-1-mediated bio-activation of proIL-1β is under the control of NLRP3 inflammasome [6]. MHV-3 infected PEMs and RAW264.7 cells exhibited with a significantly enhanced NLRP3, ASC, pro-Caspase-1 and its activated form (Caspase-1 p20) within 12h of MHV-3 infection (Fig 5A). In accordance, qPCR analyses illustrated that the mRNAs for *Nlrp3* and *proCaspase-1* were significantly higher in the virus infected livers, this correlates with observation that these virus infected livers also manifest with higher expression of the respective protein (Fig 5B). Next, we infected *Nlrp3* ^*-/-*^ mice and *Caspase-1* ^*-/-*^ mice with MHV-3 to address the importance of NLRP3 inflammasome in the causing the virus-induced liver injuries. Remarkably, a 72h viral infection largely failed to induce IL-1β expression in the livers, which was associated with significant reductions in liver FGL2 accumulation (Fig 5C), fibrinogen deposition and local tissue damages, along with significant decreases in serum ALT/AST enzymes as compared with the infected WT mice (Fig 5D). In agreement with these results, we also observed that Bgp1 expression was significantly lower in *NLRP3* ^*-/-*^ and *Caspase-1* ^*-/-*^ livers during infection (Fig 5C). Meanwhile, *NLRP3* ^*-/-*^ mice and *Caspase-1* ^*-/-*^ mice appeared to produce much less viruses at 72h of infection as compared to the WT controls (Fig 5E). Finally, *NLRP3* ^-/-^ and *Caspase-1* ^*-/-*^ mice presented with considerably prolonged survival rates toward MHV-3 infection in comparing to the WT controls (Fig 5F). The serum C5a in the viral infected *NLRP3* ^*-/-*^ and *Caspase-1* ^*-/-*^ animals was also significantly increased but no different from the WT control mice (S3B Fig), indicating that C5a up-regulation during the viral infection, appears to either additively or synergistically work with other inflammatory factors to cause viral FH. Together these observations further validate that the NLRP3/Caspase-1-inflammasome regulates the bio-processing of proIL-1β for causing the MHV-3 mediated viral FH.

**Fig 6:**
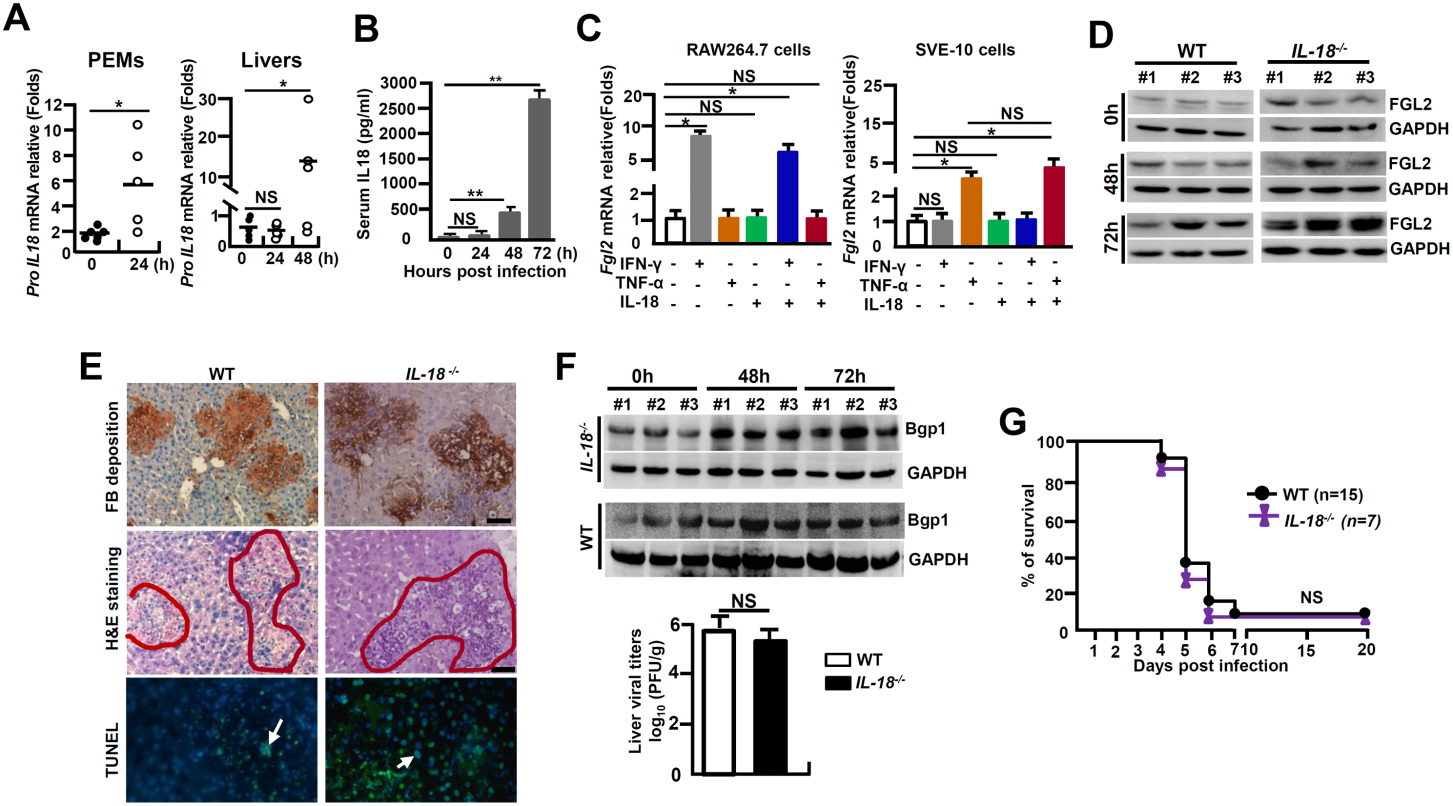
*IL-18* ^*-/-*^ mice are susceptible to MHV-3-mediated hepatitis. *IL-18* ^*-/-*^ mice and their C57BL/6 WT littermates were treated with MHV-3 (100PFU). **(A)** Peritoneal exudative macrophages (PEMs) and liver tissues were isolated from C57BL/6 WT mice, and the transcription of *proIL-18* mRNA was measured by qPCR. *p < 0.05. **(B)** Serum IL-18 levels in virus-infected WT mice at the indicated time points were measured by ELISA. **p < 0.001, n = 5~8 *per* group, NS: no significant difference. **(C)** RAW264.7 cells and SVE-10 endothelial cells were treated with IFN-γ (50 ng/ml), TNF-α (100 ng/ml) and IL-18 (20 ng/ml) alone or in combination, and *fgl2* mRNA transcription was detected by qPCR at 24h. *p < 0.05, NS: no significant difference. **(D)** The expression of FGL2 in MHV-3 infected livers was compared by western-blotting. Three representative samples *per* group are shown. **(E)** Liver fibrinogen deposition was analyzed by immunohistochemistry, architecture was detected by H&E staining, and cell apoptosis by TUNEL staining. Scale bar 20 μm, arrows indicates TUNEL-positive cells, blue color indicates nuclear staining with 4',6-diamidino-2-phenylindole (DAPI), n = 5 *per* group. **(F)** Liver expression of Bgp1 was detected by western-blot (up) and the virus titers in livers at 72h post-infection were analyzed by plaque assay, and their levels were compared by statistical analysis (down). *p<0.05, n = 5 *per* group. **(G)** The survival rate of virus-infected mice was monitored for 20 days. One of three experiments with similar results is shown. NS: no significant difference.

Assembly and activation NLRP3 inflammasome, being critical for bio-processing and activation of IL-1β, has been suggested to also involve in the bio-activation of IL-18, another member of the *IL-1* superfamily [23]. The MHV-3-infected mice showed a significant up-regulation of *proIL-18* mRNA in PEMs and livers (Fig 6A), as well as enhanced IL-18 protein in serum (Fig 6B). However, the recombinant mouse IL-18 protein (50 ng/ml) alone, or in the combination with TNF-α and INF-γ, was unable to stimulate *fgl2* mRNA transcription in RAW264.7 cells or SVE-10 endothelial cells *in vitro* (Fig 6C). Moreover, MHV-3 induced liver FGL2 production remained high in *IL-18* ^-/-^ mice (Fig 6D), showing with consequentially high levels of fibrinogen deposition, liver damages and hepatocyte necrosis (Fig 6E). Additionally, liver tissues isolated from *IL-18* ^*-/-*^ mice appear to up-regulate Bgp1 expression after MHV-3 infection. In accordance, these mice also manifested with high virus duplication (Fig 6F). Overall, *IL-18* ^-/-^ mice are still sensitive to MHV-3 infection (Fig 6G), suggesting that IL-18 is not essential in MHV-3-mediated fulminant hepatitis.

### NADPH oxidases-derived ROS triggers NLRP3 inflammasome hyperactivation in MHV-3 infected macrophages

**Fig 7:**
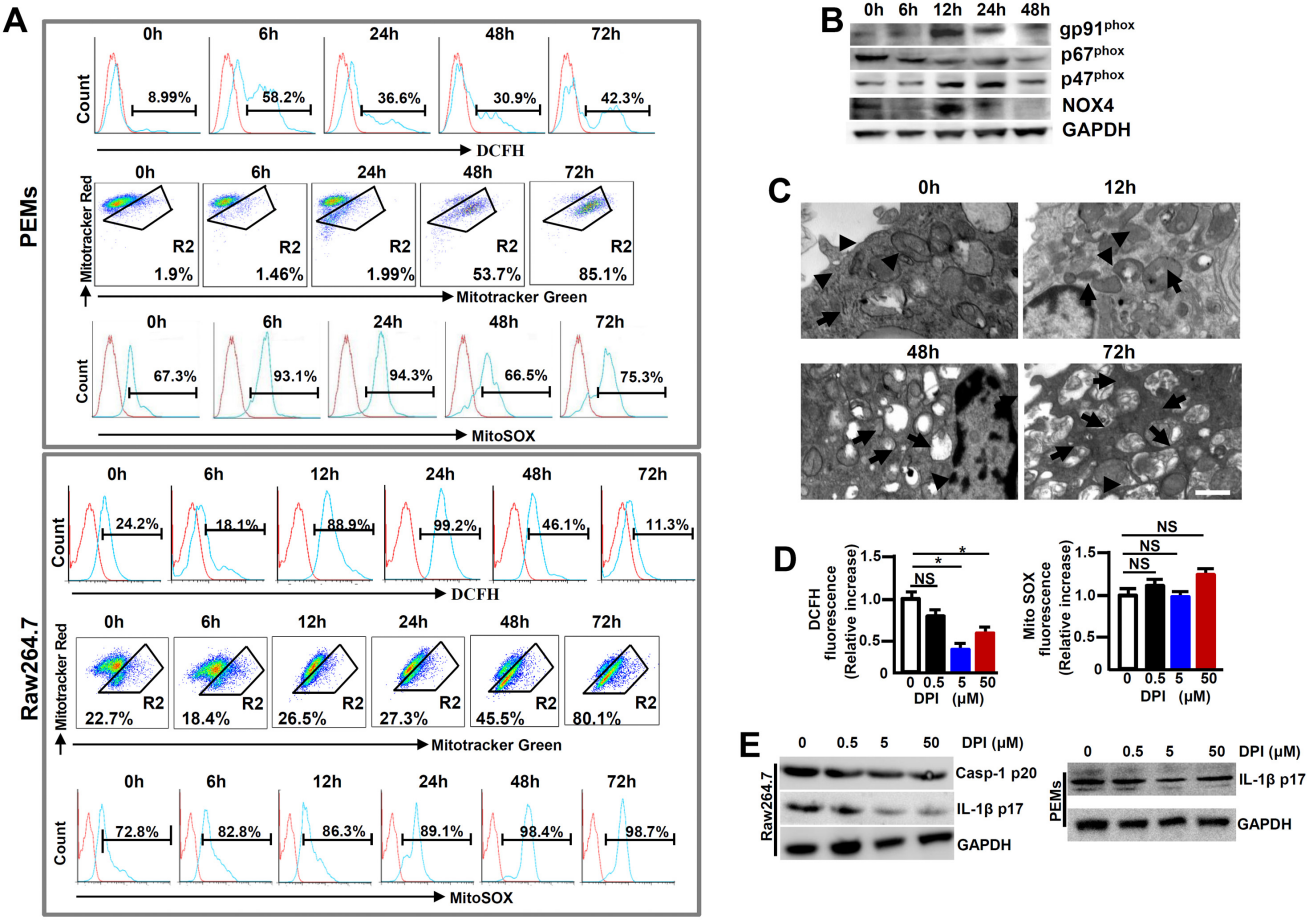
Enhanced ROS in macrophages following MHV-3 infection. PEMs and RAW264.7 cells were infected with MHV-3 (MOI = 1) *in vitro*, the NOX-derived ROS (DCFH), mitochondrial damage (stained with MitoTracker Red FM/MitoTracker Green FM) and the secretion of mitochondrial ROS (MitoSOX) were detected by flow cytometry **(A).** The gate strategies were similar to Fig 1D, and number indicates the percentage of positive cells in the gate. One of three experiments with similar results was shown. **(B)** The expression of NADPH oxidase subunits including gp91^phox^, p47^phox^ and p67^phox^ and NOX-4 in virus infected Raw264.7 cells was measured by western-blot. **(C)** Transmission electron microscopy analysis of mitochondrial morphology in virus infected cells. Arrows indicate the damaged mitochondria, whereas arrow heads indicate the normal mitochondria. (**D**) RAW264.7 cells were infected with MHV-3 for a total 24h, and cells were incubated with different doses of DPI in the last 4h. The secretion of DCFH and MitoSOX was measured by flow cytometry. Data were normalized to the increase in fluorescence of the MHV-3 infected alone sample without DPI treatment for each experiment (n = 3 independent experiments). **(E)** The expression of Caspase-1 p20 and IL-1β p17 in DPI treated RAW264.7 cells as well as PEMs was detected by western-blotting. One representative of three experiments with similar results is shown.

Many factors contribute to activating the NLRP3 inflammasome and among which, ROS is lately gaining particular attentions [13]. In order to examine the role of ROS in NLRP3 inflammasome hyperactivation, we first detected the release of NADPH oxidase-derived ROS by using a permeable dichlorohydrofluorescein (DCFH) upon MHV-3 infection. Flow cytometry showed that the releasing of DCFH from MHV-3 infected PEMs and RAW264.7 cells significantly increased, especially at 12h and 24h post-infection (Fig 7A). This result correlates with the up-regulation of gp91^phox^, p47^phox^ and NOX4, the subunits that are essential for acute ROS secretion in RAW264.7 cells (Fig 7B). However, the DCFH level dropped dramatically at 48h and 72h post the viral infection (Fig 7A), most likely owing to death of cells under these conditions (S4 Fig).

In addition to NADPH oxidase-derived ROS, mitochondria may provide an alternate source of ROS [15]. We therefore assessed the functional mitochondrial pool in MHV-3 infected cells. The viral infection in PEMs and RAW264.7 cells caused an increase in mitochondrial damage, especially at 48h and 72h post-infection, as detected by MitoTracker Green FM, a dye that stains mitochondria with no influence on their membrane potentials (Fig 7A). Similarly, electron microscopy showed with swollen mitochondria in the MHV-3 infected Raw264.7 cells at 48h and 72h (Fig 7C). This sign of mitochondrial damage seemed to strongly correlate with the increase in MitoSOX release within the same time frame (Fig 7A).

To further elucidate the role of ROS in NLRP3 inflammasome hyperactivation, we treated MHV-3 infected RAW264.7 cells with a ROS inhibitor Diphenyliodonium chloride (DPI), which is capable of preventing both NOX-dependent ROS and MitoXOS secretion [16]. NOX-originated DCFH was successfully inhibited by DPI in a dose dependent manner (Fig 7D). However, MitoXOS release was not prevented by the DPI treatment, even at a very high dose (50μM) (Fig 7D). The efficiency of NOX-originated ROS inhibition by DPI appeared to correlate with the reduction in IL-1β activation in the infected RAW264.7 cells and PEMs in dose dependent manners (Fig 7E). Together, these results suggest that the hyperactivation of NLRP3 inflammasome in macrophage is partially mediated by MHV-3 induced, NOX-derived ROS.

### *p47* ^*phox-/-*^ mice are resistance to MHV-3 induced FH by limiting NLRP3 inflammasome hyperactivation

**Fig 8:**
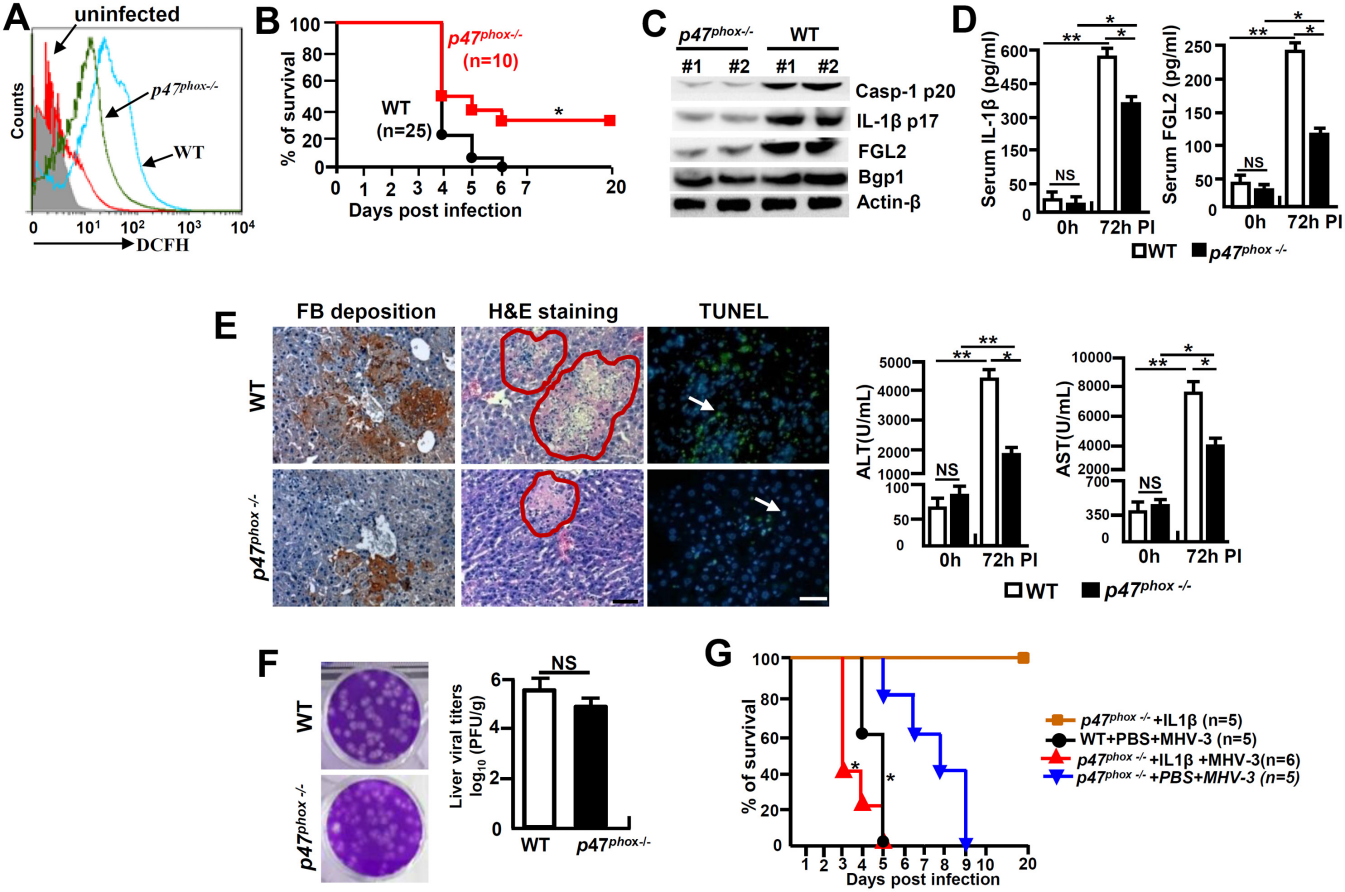
*p47* ^*phox*^ deficiency limited NLRP3 inflammasome activation and attenuated MHV-3 mediated hepatitis. The *p47* ^*phox-/-*^ mice and their C57BL/6 WT littermates were infected with MHV-3 (100PFU). **(A)** The peritoneal macrophages (PEMs) were isolated at 24h post-infection and NOX-derived ROS (DCFH) secretion was detected by flow cytometry. One representative of three experiments with similar results is shown. **(B)** The survival rate of virus infected mice was monitored. *p<0.05. One of three experiments with similar results is shown. **(C)** The expression of Casp-1 p20, IL-1β p17, Bgp1 and FGL2 at 72h post-MHV-3 infection was compared by western-blotting. **(D)** Serum IL-1β and FGL2 levels were measured by ELISA. N = 5 per group, *p<0.05 and **p<0.001, NS: no significant difference. **(E)** Liver fibrinogen deposition was analyzed by immunohistochemistry, the architecture was analyzed by H&E-staining and cellular apoptosis was analyzed using TUNEL staining (left). Scale bar 20 μm; arrow indicates positive cells; blue color indicates nuclear staining with DAPI, n = 5 *per* group. Serum ALT and AST activities were determined with an AU5400 automatic biochemistry analyzer (right). *p<0.05 and **p<0.001, n = 5 *per* group. **(F)** Liver virus titers at 72h of infection were analyzed by plaque assay (left), and their levels were compared by statistical analysis (right). *p<0.05. **(G)** MHV-3 infected *p47* ^*phox-/-*^ mice were treated with mouse recombinant IL-1β protein (100 ng/day/mouse) and the survival rate was monitored. One of three experiments with similar results is shown. *p<0.05 compared to*p47* ^*phox-/-*^ +PBS+MHV-3 group.

Cells in deficiency of *p47* ^*phox*^ exhibit a reduced capacity in generating ROS [24]. To further investigate the role of NOX-originated ROS in regulating NLRP3 inflammasome hyperactivation, we infected *p47* ^*phox-/-*^ mice with MHV-3 and examined the severity of liver pathology. As anticipated, PEMs isolated from MHV-3 infected *p47* ^*phox-/-*^ mice showed with limited DCFH (Fig 8A). Interestingly, the *p47* ^*phox-/-*^ mice also displayed considerable resistance to MHV-3 infection, presenting with reduced disease severity within the prolonged survival time as compared with the WT controls (*p* = 0.0175, Fig 8B). The lack of virus-induced ROS response, which leads to prohibition of NLRP3/caspase-1 activation and thus reduction in IL-1β production, seems to be responsible for this effect (Fig 8C and 8D). As a result, the virus infection is unable to generate significant FGL2 accumulation in the liver and serum (Fig 8C and 8D). Therefore, these mice manifested with less severe fibrinogen deposition, liver injury and hepatocyte necrosis, accompanying with low levels of AST/ALT enzymes released by the liver (Fig 8E). However, the limitation of IL-1β secretion in these *p47* ^*phox-/-*^ mice only slightly affected liver Bgp1 expression (Fig 8C), and therefore live virus titers were still high at 72h of infection (Fig 8F). Conversely, the administration of IL-1β (100 ng/mouse/day) in MHV-3 infected *p47* ^*phox-/-*^ mice was able to reinstate all aspects of disease severity typical in viral FH (Figs 8G and S5). Taken together, these results clearly indicate that the ROS/NLRP3/IL-1β axis plays a critical role in the pathogenesis of viral FH.

## Discussion

In the present work, we report that mice infected with MHV-3, an animal model for viral FH, have significantly elevated levels of IL-1β in the serum and liver. The accumulation of IL-1β accelerated liver pathology through synergistically acting with TNF-α, one of the key inflammatory cytokines that has been previously shown to be essential for causing viral FH [4, 18], *IL-1R1* signaling is responsible for stimulation of FGL2 expression in macrophages and enhancing infiltration of the inflammatory CD45^+^Gr-1^high^ neutrophils in the livers. Interestingly, MHV-3 infection in *IL-1R1* ^-/-^ mice, or in WT mice treated with IL-1β signaling inhibitors, such as using IL-1Ra, rescue the otherwise susceptible animals from the viral FH status, presenting with limited virus replication, attenuated disease progression and reduced mortality. We have also shown that the bioprocess of IL-1β maturation is under the control of a key signaling pathway, involving a MHV-3 virus inducible, ROS-dependent NLRP3 inflammasome activation. Animals lacking of *NLRP3*, *Caspase-1* or NADPH oxidase subunit *p47* ^*phox*^ that controls acute ROS secretion, all exhibited with reduced IL-1β bio-processing that results in prevention of the MHV-3 mediated disease severity. To the best of our knowledge, these data provide evidence for the first time showing that the ROS/NLRP3/IL-1β axis is an essential contributor for the virus-induced FH.

Although macrophage-mediated inflammation has been speculated to be critical for gauging the pathological susceptibility of viral FH caused by MHV-3 infection [25], the mechanisms underlying the pathogenesis are not well understood. IL-1β and IL-18 are two key inflammatory cytokines produced by macrophages which play a pivotal role in antimicrobial immunity [7, 23]. Previous studies have showed that *IL-1R1* ^*-/-*^ mice appear to have markedly reduced inflammatory pathology in the lung, presumably due to the impaired neutrophil recruitment upon influenza virus infection [26]. Conversely, Ramos *et al*. reported that *IL-1R1* ^*-/-*^ mice exhibited with a higher accumulation of the West Nile virus (WNV) in the central nervous system due to a restrained activation of the virus-specific effector CD8^+^ T cells [27]. Similarly, *IL-1β* ^*-/-*^ mice are more susceptible to herpes simplex virus 1 (HSV1)- mediated encephalitis due to an increase in viral load [28]. We here further explored the role of IL-1β in MHV-3 mediated FH. Interestingly, *IL-1R1* ^*-/-*^ animals display a significant reduction in viral duplication, amelioration of liver damage and a prolonged survival rate against MHV-3 infection (Fig 2A and 2B). These effects are probably due to *IL-1R1* deficiency lead to limit liver recruitment of CD45^+^Gr-1^high^ neutrophils and decrease in production of the macrophage-derived FGL2, which mediates sinusoidal fibrin deposition and hepatocellular necrosis in response to MHV-3 infection [3]. Bgp1 (also called carcinoembryonic cell adhesion antigen 1a,CEACAM1a) is the specific receptor for the mouse hepatitis virus (MHV), and down-regulation of Bgp1 by IFN-γ is related to the antiviral state and resistance to mouse hepatitis virus 3 infection [29]. However, Bgp1 does not appear to be involved in IL-6 and TNF-α secretion from MHV-3 infected macrophages [30]. In contrast to IFN-γ treatment, we here showed that the expression of Bgp1 drops significantly in the *IL-1R1* ^*-/-*^ liver during the viral infection, suggesting Bgp1 expression in macrophages is induced by IL-1β/IL-1R1 signaling, and lacking the pathway may compromise virus entrance and amplification. These unpredicted data implies that IL-1β has double-edge effects on the immune system, in which proper balancing with its signaling extent becomes essential for the host in protection against various invading viruses and meanwhile, in prevention of the potential collateral damage.

The molecular mechanisms that are responsible for triggering the expression of FGL2 prothrombinase, which plays a critical role in the development of MHV-3 mediated FH, are still unclear. McGilvray et al. found that both ERK and p38-MAPK proteins are activated in MHV-3 infected PEMs, and only inhibition of p38-MAPK can abolish FGL2 induction and its functional activity [31]. Jia et al. have illustrated that TNF-α upregulates FGL2 expression *via* activation of NF-kB and p38-MAPK in cardiac microvascular endothelial cells [22]. Our recent work also have showed that the inhibition of ERK1/2 and p38-MAPK efficiently block C5a-mediated FGL2 upregulation [5]. Ning et al., have demonstrated that the hepatocyte nuclear factor-4 (HNF4) cis-elements and its cognate transcription factor, HNF4α, are necessary for MHV-3-induced *fgl2* gene transcription [32]. Based on these studies, we further examined the molecular mechanisms underlying IL-1β-mediated FGL2 expression. The results show that IL-1β and TNF-α synergistically induce NF-κB, ERK and p38-MAPK tyrosine-phosphorylation (Fig 4C). However, the inhibition NF-κB pathway, but not the ERK, or p38-MAPK signals, markedly prevented FGL2 expression (Fig 4D), suggesting that the NF-κB pathways are responsible for IL-1β+TNF-α-mediated FGL2 augmentation.

The NLRP3, RIG-I and the AIM2 are three main types of inflammasome complexes that have been shown to control caspase-1 activity and IL-1β maturation. It seems that AIM2 is responsible for detecting DNA viruses, while both NLRP3 and RIG-I associate with recognition of RNA viruses by cells [33, 34]. Recent evidences suggest that the host protective immunity requires the NLRP3 inflammasome for fighting against various kinds of viruses, including Influenza A virus, modified Vaccinia virus Ankara, Sendai virus, Respiratory Syncytial virus, Encephalomyocarditis viruses, as well as Adenoviruses [35]. Our study shows that the MHV-3 triggered NLRP3, ASC and Caspase-1 mRNA as well as protein expression in PEMs and RAW264.7 cells *in vitro* (Fig 5A). Nevertheless, loss of either *NLRP3* or *Caspase-1* in macrophages reduces IL-1β secretion upon MHV-3 challenge (Fig 5C). Additionally, *NLRP3* ^*-/-*^ and *Caspase-1* ^*-/-*^ mice essentially pheno-copied the manifestations of *IL-1R1* ^*-/-*^ mice in response to MHV-3 infections, these mice evidenced with reduction in MHV-3 virus-induced IL-1β production and lessening of disease progression (Fig 5C–5F). These combined data suggest that NLRP3-inflammasome acts as a predominant pathway for triggering IL-1β maturation by MHV-3, and probably also by other corona viruses. Previous study showed that RAW264.7 cells do not release mature IL-1β because they do not express ASC [36]. Conversely, we here show that MHV-3 promotes IL-1β secretion from virus infected RAW264.7 cells through inducing ASC expression. Together with the recent work demonstrated that NLRP3/ASC/caspase-1 axis participates in the regulation of the generation of IL-1β in RAW264.7 cells, indicating that ASC is inducible in the macrophage line RAW264.7 cells under circumstances, especially during MHV-3 infection [37].

ROS plays an essential role in mediating NLRP3 inflammasome activation [13]. Many different viruses, such as Influenza virus, Respiratory Syncytial virus, and Hepatitis C virus, trigger NLRP3 inflammasome activation through ROS-dependent mechanisms [38–40]. NOX is an enzymatic complex consisting mainly of five subunits (p22^phox^, p40^phox^, p47^phox^, p67^phox^ and gp91^phox^) and two GTP-binding proteins (RAC1/RAC2). We here show that MHV-3 triggers NOX-derived ROS secretion in macrophages by inducing NOX-subunits, including GP91^phox^, p47^phox^ and NOX-4 expression in the very early stages of the viral infection (Fig 7A and 7C). Additionally, preventing NOX-derived ROS through DPI appeared to successfully down modulate NLRP3 hyperactivation and IL-1β maturation *in vitro* (Fig 7F). Furthermore, virus infected *p47* ^*phox-/-*^ macrophages manifested with significant reduction in ROS secretion, leading to the control of NLRP3 hyperactivation, which results in attenuation in severity of the viral FH (Fig 8). These results are inconsistent with previous reports that have shown that NADPH oxidase-derived ROS are not involved in activating NLRP3 inflammasome [41, 42]. One of the discrepancies is the different cell models are used in studies. Silica crystals, LPS, and uric acid crystals act as the stimulators in these studies, while MHV-3 virus is the activator in our research. Conversely, it is worth mentioning that not all *p47* ^*phox-/-*^ mice are completely resistant to MHV-3, and these animals eventually still died from the infections (Fig 8B), together with some virus infected mice still produce high levels of IL-1β and virus titers, suggesting the presence of other mediators that in response to the virus challenge, are capable of activating NLRP3 inflammasome *in vivo*. One of the potential activators is MitoSOX [43, 44]. We have also observed a very high level of the MitoSOX production in the MHV-3 infected RAW264.7 cells at 48h and 72h post-infection *in vitro*, along with high frequency damage and destruction of mitochondria might simultaneously occur. However, the release of MitoSOX was unable to be successfully blocked by ROS inhibitor- DPI (50 uM) (Fig 7). Additionally, DPI is harmful to animals and unsuitable *in vivo* experiments [45]. The incapable of completely blocking ROS production by using high dose of DPI *in vitro* suggests the existence of other sources of ROS for activating NLRP3 inflammasome. Interestingly, reduced mortality and pathology were seen in MHV-3 infected *p47* ^*phox-/-*^ mice compared to WT littermates despite a lack of significant reduction in virus replication, suggesting that MHV-3-mediated pathology is due to inflammation and not direct virus infection. Recent studies by Warner Greene’s group demonstrate that HIV can trigger caspase-1 activation and pyroptosis, a highly inflammatory form of programmed cell death in which dying cells release their cytoplasmic contents, including inflammatory cytokines into the extracellular space where the virus infected CD4^+^ T-cells recite [46]. A similar environment might also explain for the MHV-3 induced FH status.

IL-18 is another member of the IL-1 superfamily that has been indicated to be important in the pathogenesis of mouse models of Influenza virus, HBV, Rhinovirus and Vaccinia virus infection [47]. For example, *IL-18R* ^*-/-*^ mice appeared to be protected from Influenza viral initiated inflammatory lung damages [48]. Consistent with previous reports, we have detected significantly high levels of matured IL-18 in the serum of MHV-3 infected WT mice. However, *IL-18* deficiency does not prevent Bgp1 expression, virus amplification and FGL2 accumulation in the liver following MHV-3 infection, and as the consequence, these mice stay high with fibrinogen deposition, liver damage and hepatocyte necrosis (Fig 6). These results suggest that IL-18 is not essential for causing MHV-3 mediated acute hepatitis.

In conclusion, our study elucidates that NLRP3 inflammasome-dependent IL-1β production, a primary inflammatory signaling pathway of the host for mounting conventional immunity against pathogen invasions, plays a double-edged role in the host immune system. Hepatotropic virus, like MHV-3 infection in mice, can induce exaggerated inflammation in the liver and cause life-threatening viral FH. These results shed lights on a novel strategy, for which the properly modulation of the IL-1β signaling pathway, in combination with blocking other inflammatory factors, might benefit the treatment of viral FH and other severe inflammatory diseases in human.

## Materials and Methods

### Mice

The *p47* ^*phox*^-deficient (*p47* ^*phox-/-*^, #004742), *NLRP3* ^*-/-*^ (#017970), *Caspase-1* ^*-/-*^ (#016621), *IL-18* ^*-/-*^ (#004130), *IL-1R1* ^*-/-*^ (#003245) and wild type (WT) mice were on C57BL/6 background and were purchased from the Jackson Laboratory (Bar Harbor, Maine, USA). Mice were maintained in micro-isolator cages, fed with standard laboratory chow diet and water, and housed in the animal colony at the animal center of the Third Military Medical University (TMMU). Mice approximately 12 weeks of age were used for these experiments. All animals received humane care according to the criteria outlined in the "Guide for the Care and Use of Laboratory Animals" prepared by the National Academy of Sciences and published by the National Institutes of Health (NIH publication 86–23 revised 1985).

### Cells

RAW264.7 cells were provided by the Cell Institute of the Chinese Academy of Sciences (Shanghai, China). Peritoneal exudative macrophages (PEMs) were harvested as described previously [5]. Cells were cultured in 6-well plates and propagated in DMEM supplemented with 10% FBS, 100 U/ml penicillin, and 100 μg/ml streptomycin.

### Virus and infection

MHV-3 viruses were expanded in murine 17CL1 cells to a concentration of 1×10^7^ plaque forming unit (PFU)/ml. The virus-containing supernatants were stored at -80°C until use. Macrophages were infected with MHV-3 (multiplicity of infection, MOI = 1) *in vitro* and mice were injected with 100 PFU of MHV-3 *via i*.*p*. In some experiments, the virus infected mice were treated with IL-1R antagonist (IL-1Ra, 10 mg/kg/day) or recombinant mouse IL-1β protein (100 ng/day/mouse) every day. Mice were euthanized on the indicated days and the virus titers in liver were determined by plaque assay as described previously [25]. The sources of antibodies and other reagents are detailed in S1 Text.

### Tissue morphology detection and immunohistochemistry

Paraffin-embedded liver tissue blocks were cut into 3 μm slices and mounted onto poly-lysine-charged glass slides, and tissue injury was stained by hematoxylin and eosin (H&E). Cellular apoptosis was measured by TUNEL staining according to the manufacturer's instructions (Roche, Berlin, Germany). The expression of fibrinogen and FGL2 was detected by immunohistochemistry as described previously [25]. Sections were scored in a blinded fashion for histological diagnosis.

### Real-time quantitative RT-PCR

Total RNA was extracted from cultured cells or liver tissues with TRIzol reagent according to the manufacturer's instructions (Invitrogen, NY, USA). First-strand cDNA was synthesized with the PrimeScript RT-PCR Kit (Takara, Dalian, China). The expression of mRNA encoding for *NLRP3*, *Caspase-1*, *proIL-1β* and *proIL-18* was quantified by real-time quantitative PCR with the SYBR Premix ExTaq kit (Takara) and was normalized to the expression of β-actin. Sequences of the primers are provided in S1 Table. Results were calculated and compared by the 2^−ΔΔCt^ method.

### ELISA and western-blotting

Serum C5a, FGL2, IL-18 and IL-1β levels were measured by ELISA. The expression of FGL2, proCaspase-1, Caspase-1-p20, NLRP-3, p47^phox^, p90^phox^, p67^phox^, Nox-4, Bgp1, proIL-1β and IL-1β-p17 in MHV-3 infected livers or macrophages was detected by western-blotting described previously [25].

### Flow cytometry

The release of IL-1β/ROS from virus infected macrophages, liver infiltration of CD45^+^GR-1^high^ neutrophil and CD4^+^Foxp3^+^ regulatory T cells (Treg), all were detected by flow cytometry (FACsAria cytometer, BD, Franklin Lakes, NJ, USA). The death cells were excluded firstly by staining with LIVE/DEATH Fixable Near-IR Ded Cell Stain Kit (Life technologies, Eugene, Oregon, USA). The secretion of NOX-derived ROS was detected by means of an oxidation-sensitive fluorescent probe-DCFH according to the manufacturer's instructions (Beyotime, Shanghai, China). Moreover, the mitochondria-derived ROS was measured in cells stained with MitoSOX (5 μM, Invitrogen) for 20 min. To measure mitochondrial damage, cells were stained for 20 min with MitoTracker Green FM (20 nM) and MitoTracker Deep Red FM (20 nM), two kinds of dye that stain mitochondria with no influence on their membrane potentials (Invitrogen). A total of 10,000 live cells were analyzed. All the FACs data were analyzed using CellQuest Pro software.

### Electron microscopy

RAW264.7 cells or primary PEMs isolated from MHV-3 infected mice were fixed with 4% (v/v) glutaraldehyde. Sample preparation was conducted as described previously [49]. Mitochondrial morphology and virion was observed with JEOL JEM2100HC transmission electron microscopy.

### Statistical analysis

All data were analyzed using GraphPad Prism 4.03 software. An unpaired Student’s *t*-test (two-tailed) was used to assess comparisons between two groups when the data met the assumptions of the *t*-test. Survival curves were generated by log-rank test. *p*<0.05 was considered a significant difference.

### Ethics statement

All animal experiments were performed in strict accordance with the Guide for the Care and Use of Laboratory Animals issued by the Ministry of Science and Technology of the People's Republic of China. The protocol was approved by the Third Military Medical University Institutional Animal Care and Use Committee.

## Supporting Information

S1 Text Reagents and antibodies. (DOCX)

S1 Table The primer sequences for qPCR of the indicated genes. (DOCX)

S1 Fig IL-1R antagonist (IL-1Ra) protected MHV-3-mediated hepatitis. C57BL/6 WT mice were infected with MHV-3 (100 PFU) and treated with IL-1R antagonist (IL-1Ra, 10 mg/kg/day) or PBS at the same time. **(A)** Serum IL-1β concentration at 72h post MHV-3 infection was measured by ELISA. **p<0.001. **(B)** Liver Bgp1 and FGL2 expression at 72h post-infection was detected by western-blotting. **(C)** The virus titers in livers at 72h post-infection were analyzed by plaque assay, and their levels were compared by statistical analysis. *p<0.05, n = 5 *per* group. **(D)** Liver architecture was analyzed by H&E-staining, the FGL2 expression and fibrinogen deposition was analyzed by immunohistochemistry. N = 5 *per* group, scale bar = 20 μm. **(E)** The survival rate was monitored for a total of 20 days. One representative of three experiments with similar results is shown.*p<0.05 compared to MHV-3+PBS group.
(TIF)

S2 Fig Liver infiltration of CD4^+^Foxp3^+^ Tregs was not affected in *IL-1R1* ^*-/-*^ mice. *IL-1R1* ^*-/-*^ mice and their C57BL/6 WT littermates were infected with MHV-3 (100 PFU). **(A)** The percentage of CD4^+^Foxp3^+^ Tregs in liver tissue was detected by flow cytometry. One representative of five mice *per* group is shown. The number indicates the percentage of positive cells in the indicated gate. **(B)** The number of CD4^+^Foxp3^+^ Tregs in liver tissues was counted and compared. *p < 0.05. NS: no significant difference.
(TIF)

S3 Fig Serum C5a concentration was not affected by IL-1β and NLRP3 inflamasome. *IL-1R1* ^*-/-*^, *NLRP3* ^*-/-*^, *Caspase-1* ^*-/-*^ mice and their C57BL/6 WT littermates were infected with MHV-3 (100 PFU). **(A)** Serum complement C5a concentration between *IL-1R1* ^*-/-*^ and WT mice was measured by ELISA and statistically compared. N = 6 *per* group. N = 6 *per* group, **p < 0.001. NS: no significant difference. **(B)** Serum complement C5a concentration among *NLRP3* ^*-/-*^, *Caspase-1* ^*-/-*^ mice and their WT littermates was measured by ELISA and statistically analyzed. N = 6 *per* group,**p < 0.05. NS: no significant difference.
(TIF)

S4 Fig MHV-3 promotes RAW264.7 cell apoptosis. RAW264.7 cells were infected with MHV-3 (MOI = 1), and cellular apoptosis was analyzed using TUNEL staining at the indicated time points. Scale bar 20 μm; arrow indicates positive cells; blue color indicates nuclear staining with DAPI.
(TIF)

S5 Fig Restoration of IL-1β exacerbates MHV-3-mediated hepatitis in *p47* ^*phox-/-*^ mice. MHV-3-infected *p47* ^*phox-/-*^ mice were treated with mouse recombinant IL-1β protein (100 ng/day/mouse) or PBS, respectively. **(A)** Serum FGL2 levels post-infection were measured by ELISA (n = 5 per group). *p<0.05. NS: no significant difference. **(B)** Liver fibrinogen (FB) deposition at 72h post-infection was detected by immunohistochemistry and the architecture was analyzed by H&E-staining. N = 5 *per* group, scale bar 20 μm, arrow indicates positive cells.
(TIF)
