# Correction: The NLRP3 Inflammasome and IL-1β Accelerate Immunologically Mediated Pathology in Experimental Viral Fulminant Hepatitis

**DOI:** 10.1371/journal.ppat.1005216

**Published:** 2015-10-09

**Authors:** Sheng Guo, Chengying Yang, Bo Diao, Xiaoyong Huang, Meihua Jin, Lili Chen, Weiming Yan, Qin Ning, Lixin Zheng, Yuzhang Wu, Yongwen Chen

The Funding statement is incorrect. The correct Funding statement is:

This work was supported by The General Program of National Natural Science Foundation of China (NSFC, No.81361120400, 81222023, 81471949 and 81171585). The funders had no role in study design, data collection and analysis, decision to publish, or preparation of the manuscript.

## References

[1] Guo S, Yang C, Diao B, Huang X, Jin M, Chen L, et al (2015) The NLRP3 Inflammasome and IL-1β Accelerate Immunologically Mediated Pathology in Experimental Viral Fulminant Hepatitis. PLoS Pathog 11(9): e1005155 doi: 10.1371/journal.ppat.1005155 26367131 10.1371/journal.ppat.1005155 PMC4569300

