# Correction: The NLRP3 Inflammasome and IL-1β Accelerate Immunologically Mediated Pathology in Experimental Viral Fulminant Hepatitis

**DOI:** 10.1371/journal.ppat.1005406

**Published:** 2016-02-29

**Authors:** PLOS Pathogens Staff

Fig 3 is incorrect. The authors have provided a corrected version here. The publisher apologizes for the error.

**Fig 3:**
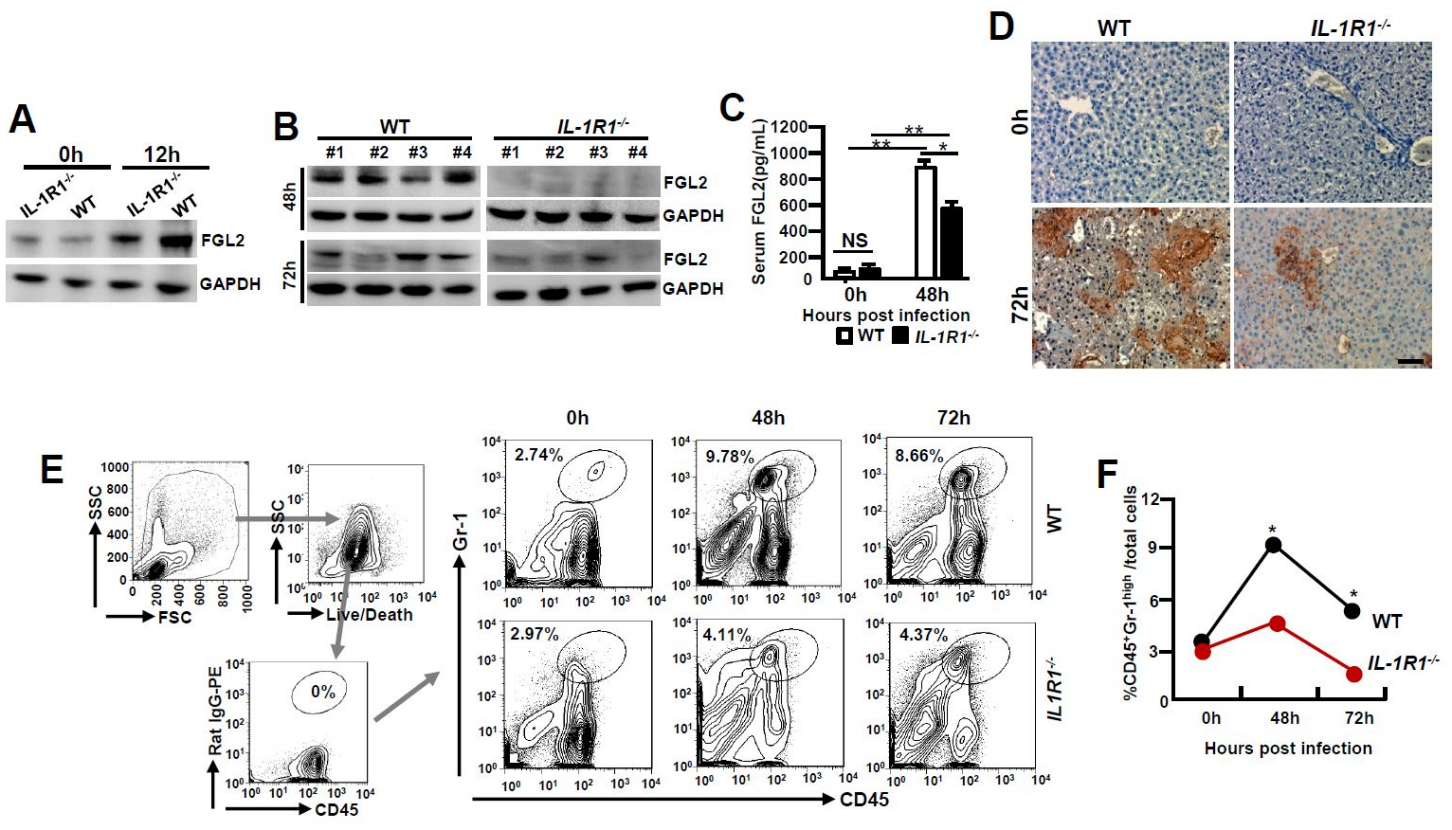
MHV-3 fails to induce FGL2 production and neutrophil infiltration in the livers of *IL-1R1-/-* mice. *IL-1R1-/-* mice and their C57BL/6 WT littermates were infected with MHV-3 (100 PFU). (A) Peritoneal exudative macrophages (PEMs) were isolated and the expression of FGL2 was detected by western-blotting. (B) The expression of FGL2 in liver at 48h and 72h post-infection was analyzed by western-blotting. Four representative samples *per* group are shown. (C) Serum FGL2 levels in virus infected mice were measured by ELISA.*p<0.05 and **p<0.0001, NS: no significant difference, n = 5 *per* group. (D) The liver fibrinogen deposition post-infection was analyzed by immunohistochemistry. Scale bar 20 μm, n = 6~8 per group. (E) Liver recruitment of CD45+Gr-1^high^ neutrophils after MHV-3 infection was measured by flow cytometry. The left panels are gate strategies, and number indicates the percentage of positive cells in the gate. One representative sample from five mice *per* group is showed. (F) Statistical analysis of liver CD45+Gr-1^high^ neutrophil infiltration. *p<0.05 compared to WT littermates in each group, n = 5 *per* group.
