# Supplementary material for: The NLRP3 Inflammasome and IL-1β Accelerate Immunologically Mediated Pathology in Experimental Viral Fulminant Hepatitis
Source: PLoS Pathog. 2015 Sep 14;11(9):e1005155. doi: 10.1371/journal.ppat.1005155 (PMC4569300; doi:10.1371/journal.ppat.1005155)
Supplement: S1 Text — (DOCX) [file ppat.1005155.s001.docx]

**Supporting information**

**S1 Text**

**Reagents and antibodies** [Diphenyleneiodonium chloride](http://www.sigmaaldrich.com/catalog/product/sigma/d2926?lang=zh&region=CN) (DPI), 4,6-diamidino-2-phenylindole (DAPI) and protease inhibitor 'cocktail' were from Sigma-Aldrich. Recombinant mouse IL-1β and TNF-α were purchased from PeproTech (Rocky Hill, NJ, USA). Phycoerythrin (PE)-conjugated antibody to mouse F4/80 (#12-4801), fluorescein isothiocyanate (FITC)-conjugated antibody to mouse IL-1β (#11-7114), PE-anti-Gr-1 (#12-5931), FITC-anti-CD45 (#8011-9459), mouse regulatory T cell staining kit #1 (#88-8111), the human IL-1Ra (#0509474), mouse IL-1α[Platinum ELISA](http://www.ebioscience.com/mouse-il-1beta-platinum-elisa-kit.htm) (BMS611), mouse IL-1β [Platinum ELISA](http://www.ebioscience.com/mouse-il-1beta-platinum-elisa-kit.htm) (BMS6002), and mouse IL-18 [Platinum ELISA](http://www.ebioscience.com/mouse-il-1beta-platinum-elisa-kit.htm) (#BMS618/3) Kits were from eBioscience (San Diego, CA USA). Antibody to IκBα (44D4) and IκBα phosphorylated at Ser32 (14D4), antibody to Erk (#9102) and Erk phosphorylated at Thr202/Tyr204 (#9101) were from Cell Signaling Technology (Boston, MA, USA). Antibody to NF-κBp65 phosphorylated at Ser276 (#J0212), anti-NF-κBp65 (#A2813), antibody to p38 phosphorylated at Tyr182 (#J0212), anti-P38 (#E0614), anti-P47^phox^ (#H0111), anti-P67^phox^ (#E1613), anti-GP91^phox^ (#B1214), anti-NOX4 (#L0413), anti-NLRP3 (#3113), anti-FGL2 (#C1009), anti-Bgp1 (#L2210), anti-ProCaspase-1 (#B2013), anti-Caspase-1p20 (#B0413), anti-proIL-1β (#B2013), **anti-ASC (sc-22514-R), a**nti-GAPDH and anti-Actin-β were from Santa Cruz (San Diego, CA USA). Anti-IL-1βp17 (#ab9722) was from Abcam (Cambridge, UK). **Anti-NLRP3 (AG-20B-0014-C100) was from Adipogen (San Diego, CA, USA).** TRIzol reagents were from Invitrogen **(**Burlingame, CA, USA**)**. The PrimeScript RT-PCR Kits and SYBR Premix ExTaq kits were from Takara Bio (Dalian, China). A Terminal TransferasedUTP Nick End Labeling (TUNEL) staining kit was from Roche (Mannheim, Germany).Mouse complement C5a ELISA Kit(#[EK0987](http://www.boster.com.cn/Item/5098.aspx)) was supplied by Boster Biotech. Ltd. (Wuhan, China).
