# Supplementary material for: The NLRP3 Inflammasome and IL-1β Accelerate Immunologically Mediated Pathology in Experimental Viral Fulminant Hepatitis
Source: PLoS Pathog. 2015 Sep 14;11(9):e1005155. doi: 10.1371/journal.ppat.1005155 (PMC4569300; doi:10.1371/journal.ppat.1005155)
Supplement: S1 Table — (DOCX) [file ppat.1005155.s002.docx]

**Supporting information**

**S1 Table The primer sequences for qPCR of the indicated genes**

| Gene name | Sense primer sequence | Anti-sense primer sequence |
| --- | --- | --- |
| *fgl2* | 5`-TGGACAACAAAGTGGCAAATCT-3` | 5`-TGGAACACTTGCCATCCAAA-3` |
| *proIL-1β* | 5' -CAGGCAGGCAGTATCACTCATTG-3` | 5' -CGTCACACACCAGCAGGTTATC-3` |
| *proIL-18* | 5' -TGAAGTAAGAGGACTGGCTGTGAC-3` | 5' -ATCTTGTTGTGTCCTGGAACACG-3` |
| *β-actin* | 5'-CACTATCGGCAATGAGCGGTTCC-3` | 5'-CAGCACTGTGTTGGCATAGAGGTC-3` |
| *Caspase-1* | 5'-AAGAACAGAACAAAGAAGATGGCACA-3` | 5'- ACCCTCGGAGAAAGATGTTGAAA-3` |
| *NLRP-3* | 5'-CCTGACCCAAACCCACCAGT-3` | 5'-TTCTTTCGGATGAGGCTGCTTA-3` |
