# Supplementary figures and images for: The NLRP3 Inflammasome and IL-1β Accelerate Immunologically Mediated Pathology in Experimental Viral Fulminant Hepatitis
Source: PLoS Pathog. 2015 Sep 14;11(9):e1005155. doi: 10.1371/journal.ppat.1005155 (PMC4569300; doi:10.1371/journal.ppat.1005155)

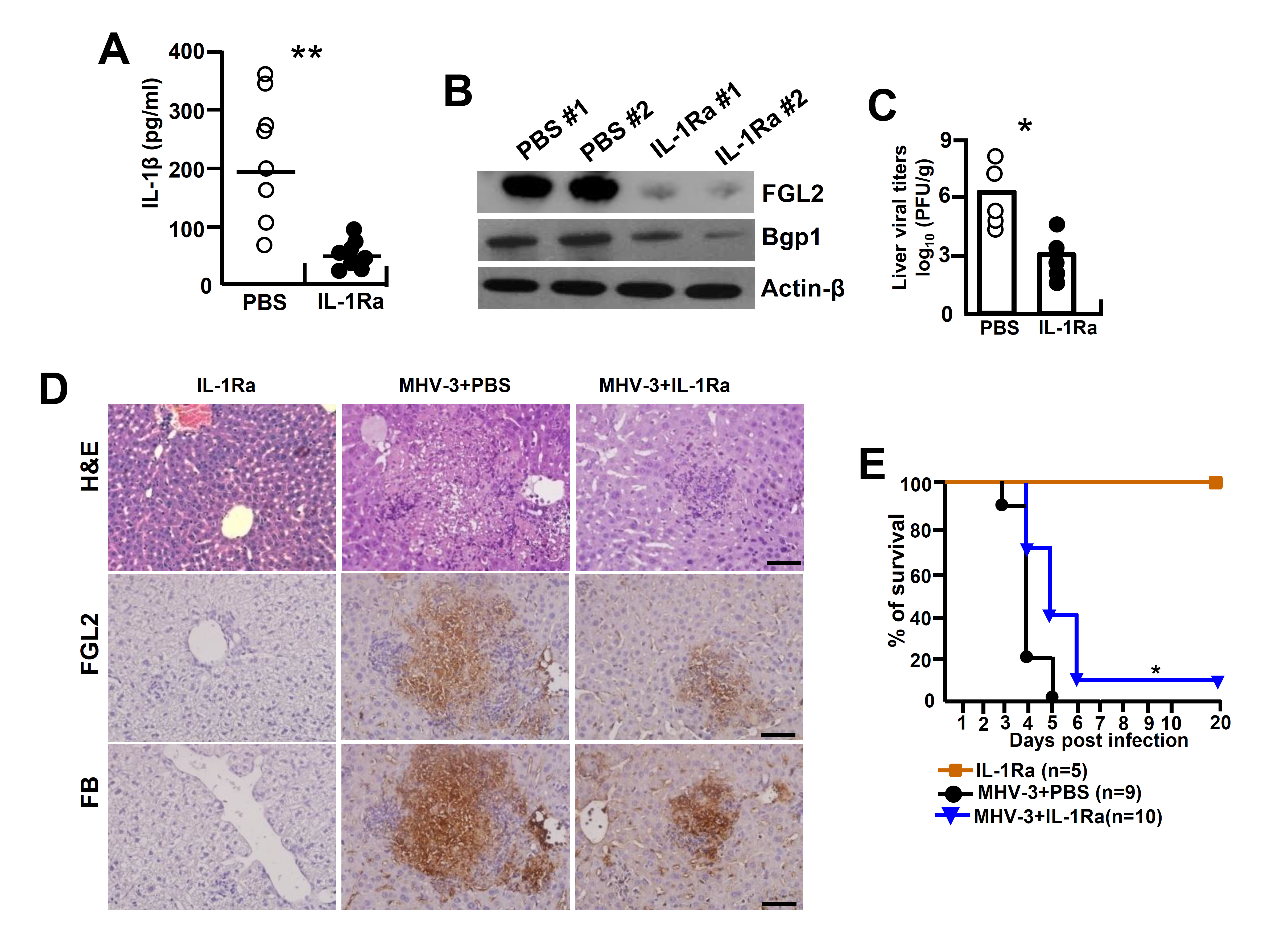

Supplement: S1 Fig — C57BL/6 WT mice were infected with MHV-3 (100 PFU) and treated with IL-1R antagonist (IL-1Ra, 10 mg/kg/day) or PBS at the same time. (A) Serum IL-1β concentration at 72h post MHV-3 infection was measured by ELISA. **p<0.001. (B) Liver Bgp1 and FGL2 expression at 72h post-infection was detected by western-blotting. (C) The virus titers in livers at 72h post-infection were analyzed by plaque assay, and their levels were compared by statistical analysis. *p<0.05, n = 5 per group. (D) Liver architecture was analyzed by H&E-staining, the FGL2 expression and fibrinogen deposition was analyzed by immunohistochemistry. N = 5 per group, scale bar = 20 μm. (E) The survival rate was monitored for a total of 20 days. One representative of three experiments with similar results is shown.*p<0.05 compared to MHV-3+PBS group. (TIF) [file ppat.1005155.s003.tif]

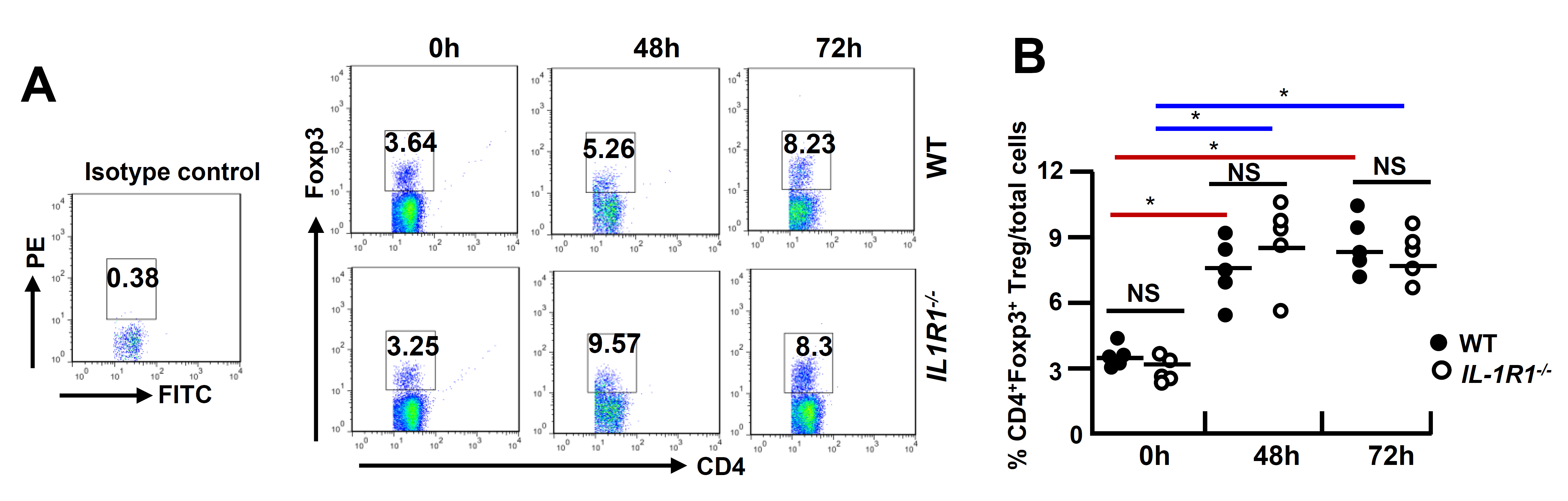

Supplement: S2 Fig — IL-1R1 -/- mice and their C57BL/6 WT littermates were infected with MHV-3 (100 PFU). (A) The percentage of CD4+Foxp3+ Tregs in liver tissue was detected by flow cytometry. One representative of five mice per group is shown. The number indicates the percentage of positive cells in the indicated gate. (B) The number of CD4+Foxp3+ Tregs in liver tissues was counted and compared. *p < 0.05. NS: no significant difference. (TIF) [file ppat.1005155.s004.tif]

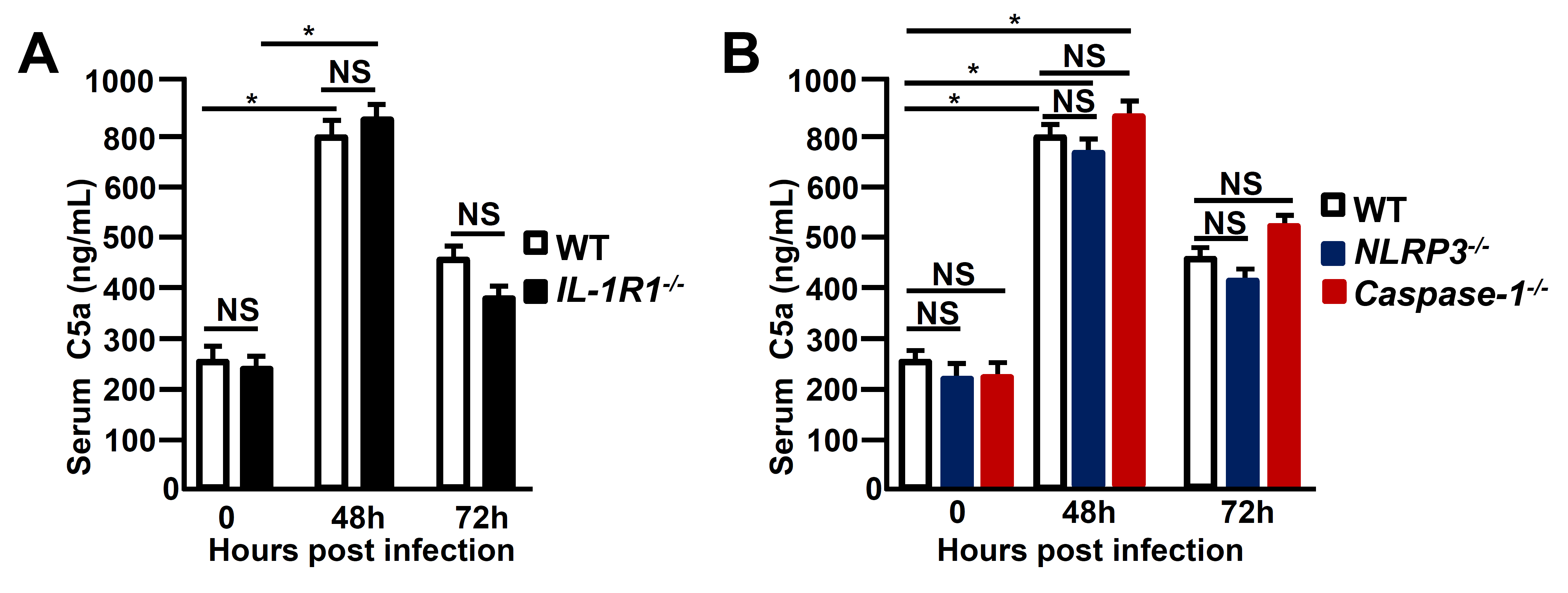

Supplement: S3 Fig — IL-1R1 -/-, NLRP3 -/-, Caspase-1 -/- mice and their C57BL/6 WT littermates were infected with MHV-3 (100 PFU). (A) Serum complement C5a concentration between IL-1R1 -/- and WT mice was measured by ELISA and statistically compared. N = 6 per group. N = 6 per group, **p < 0.001. NS: no significant difference. (B) Serum complement C5a concentration among NLRP3 -/-, Caspase-1 -/- mice and their WT littermates was measured by ELISA and statistically analyzed. N = 6 per group,**p < 0.05. NS: no significant difference. (TIF) [file ppat.1005155.s005.tif]

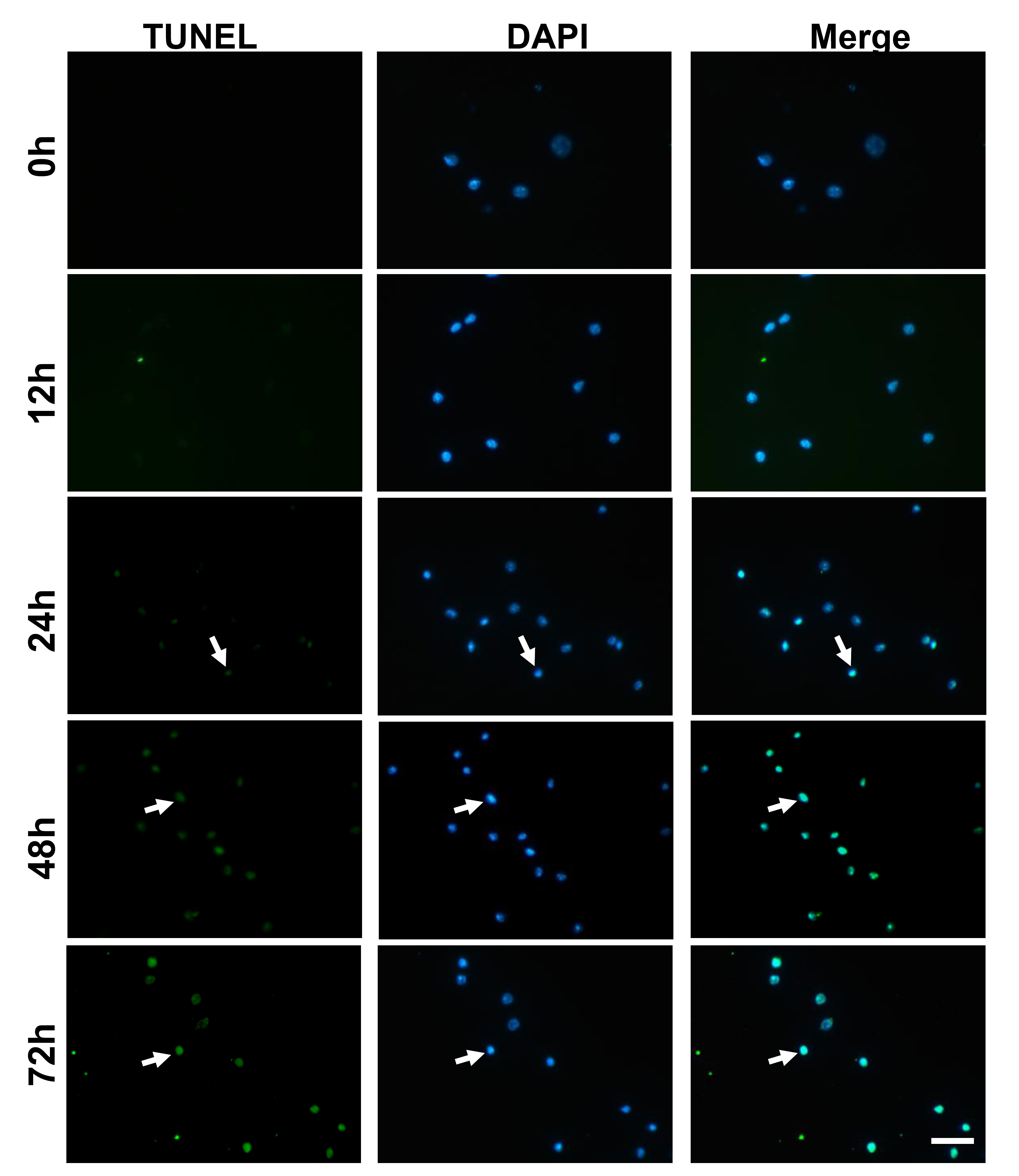

Supplement: S4 Fig — RAW264.7 cells were infected with MHV-3 (MOI = 1), and cellular apoptosis was analyzed using TUNEL staining at the indicated time points. Scale bar 20 μm; arrow indicates positive cells; blue color indicates nuclear staining with DAPI. (TIF) [file ppat.1005155.s006.tif]

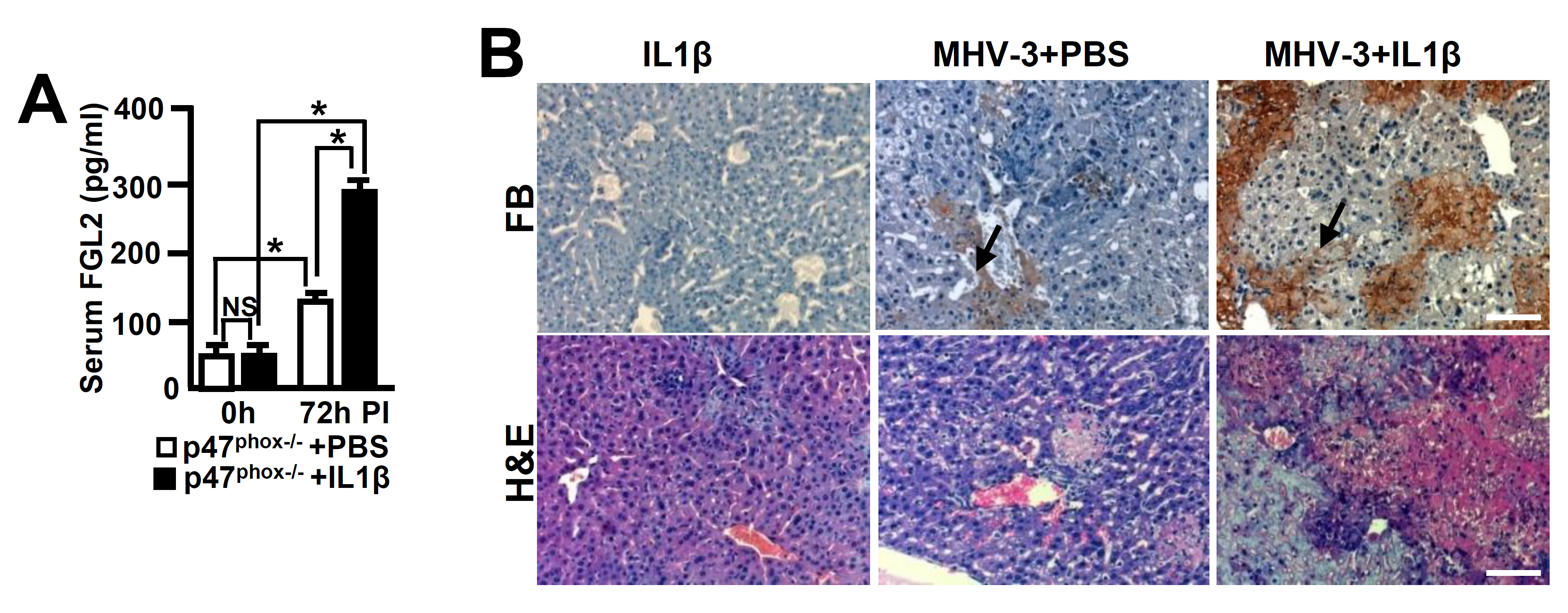

Supplement: S5 Fig — MHV-3-infected p47 phox-/- mice were treated with mouse recombinant IL-1β protein (100 ng/day/mouse) or PBS, respectively. (A) Serum FGL2 levels post-infection were measured by ELISA (n = 5 per group). *p<0.05. NS: no significant difference. (B) Liver fibrinogen (FB) deposition at 72h post-infection was detected by immunohistochemistry and the architecture was analyzed by H&E-staining. N = 5 per group, scale bar 20 μm, arrow indicates positive cells. (TIF) [file ppat.1005155.s007.tif]
